# Restoring miR122 in human stem-like hepatocarcinoma cells, prompts tumor dormancy through Smad-independent TGF-β pathway

**DOI:** 10.18632/oncotarget.11885

**Published:** 2016-09-07

**Authors:** Loreto Boix, Juan Manuel López-Oliva, Ana Carolina Rhodes, Jordi Bruix

**Affiliations:** ^1^ Barcelona Clinic Liver Cancer (BCLC) Group, Liver Unit, Hospital Clínic of Barcelona, University of Barcelona, Institut d'Investigacions Biomèdiques August Pi i Sunyer (IDIBAPS), Fundació Clínic per a la Recerca Biomèdica (FCRB), CIBERehd, 08036 Barcelona, Spain

**Keywords:** hepatocellular carcinoma, miR122, cancer stem cell, tumor initiating cell, tumor dormancy

## Abstract

miR122 is the prevalent miRNA in adult healthy liver and it is responsible for liver stem cell differentiation towards hepatocyte lineage. Its expression is frequently lost in hepatocellular carcinoma (HCC). We studied the effects of restoring miR122 expression in a distinctive cell line derived from human HCC-BCLC9 cells-with a solid stem-like cell profile, high tumor initiating ability and undetectable miR122 expression. We generated a stable BCLC9 cell line that expresses miR122 (BCLC9-miR122). Restitution of miR122 in BCLC9 cells, decreases cell proliferation rate and reduces significantly tumor size *in vivo*. BCLC9-miR122 cells down-regulate expression of *MYC, KLF4, FOXM1, AKT2* and *AKT3* genes and up-regulate *FOXO1* and *FOXO3A* gene expression. In addition, miR122 transfected cells decreased AKT2 kinase activation while decreased FOXO1 and FOXO3A protein inactivation. Reduction in tumor size in BCLC9-miR122 associated with an increase in p38MAPK protein expression and activation leading to a low phospho-ERK1/2 to phospho-p38 ratio. Treatment of miR122 positive cells with an inhibitor of TGFBR1 activation, abolished tumor dormancy program and recovered cell proliferation rate through a Smad-independent TGF-β response.

HCC stem-like cells can be directed towards cell differentiation and tumor dormancy by restoring miR122 expression. We demonstrate, for the first time, that dormancy program is achieved through a Smad-independent TGF-β pathway. Reestablishing miR122 expression is a promising therapeutic strategy that would work concurrently reducing tumor aggressiveness and decreasing disease recurrence.

## INTRODUCTION

MicroRNAs (miRs) are regulators of gene expression by destabilizing and inducing degradation of messenger RNAs (mRNA) and/or repressing their translation [1]. miRs are key in cancer biomarker research due to their tissue specificity [2]. For instance, miR122 accounts for 72% of all miRs in the liver and it is undetectable in all other tissues analyzed [3]. Besides the well-known role of miR122 in favoring hepatitis C virus replication in hepatocytes [4], its function in the normal adult liver is related to cholesterol and lipid homeostasis [5]. miR122 expression increases along with liver development. It reaches its maximum levels in adult healthy liver and is associated with hepatocyte differentiation. Up to 70% of hepatocellular carcinomas (HCC) show miR122 down-regulation [6] and the same applies for HCC-derived cell lines [7]. Several studies forcing expression of miR122 in HCC cell lines, describe miR122 as a liver tumor suppressor [8] and hepatocyte cell differentiation factor [9].

HCC remains one of the leading causes of cancer-related death in the world [10]. Despite the improvement in patient prognosis due to earlier diagnosis and new treatment strategies, long term effective therapy for advanced disease and prevention of tumor recurrence are still an unmet need [11]. This is probably favoured by the presence of a subpopulation of transformed cells with high tumor initiating ability that are known as cancer stem cells (CSCs) or tumor initiating cells (TICs). BCLC9 is a unique cell line derived from human HCC [12] that exhibits a wide range of stem cell markers, self-renewal ability, they usually grow forming spheroid structures on standard plastic culture plates and they show high tumor-initiating potential. So, as far as we know, this is the unique human hepatocarcinoma cell line with a well-founded cancer stem cell phenotype. Since no other human HCC cell line offers this profile, there is no possibility to verify our results in other liver cell line although effects of miR122 on differentiation of non-transformed progenitor cells are well reported. Accordingly, restoring miR122 expression in HCC cells with CSC phenotype will depict the mechanisms involved in the tumor suppressor impact of miR122 and it will provide valuable information about potential therapeutic targets for refractory/recurrent HCC.

## RESULTS

### miR122 increases adherence of cell spheroids and down-regulates pluripotency markers *in vitro*

**Figure 1:**
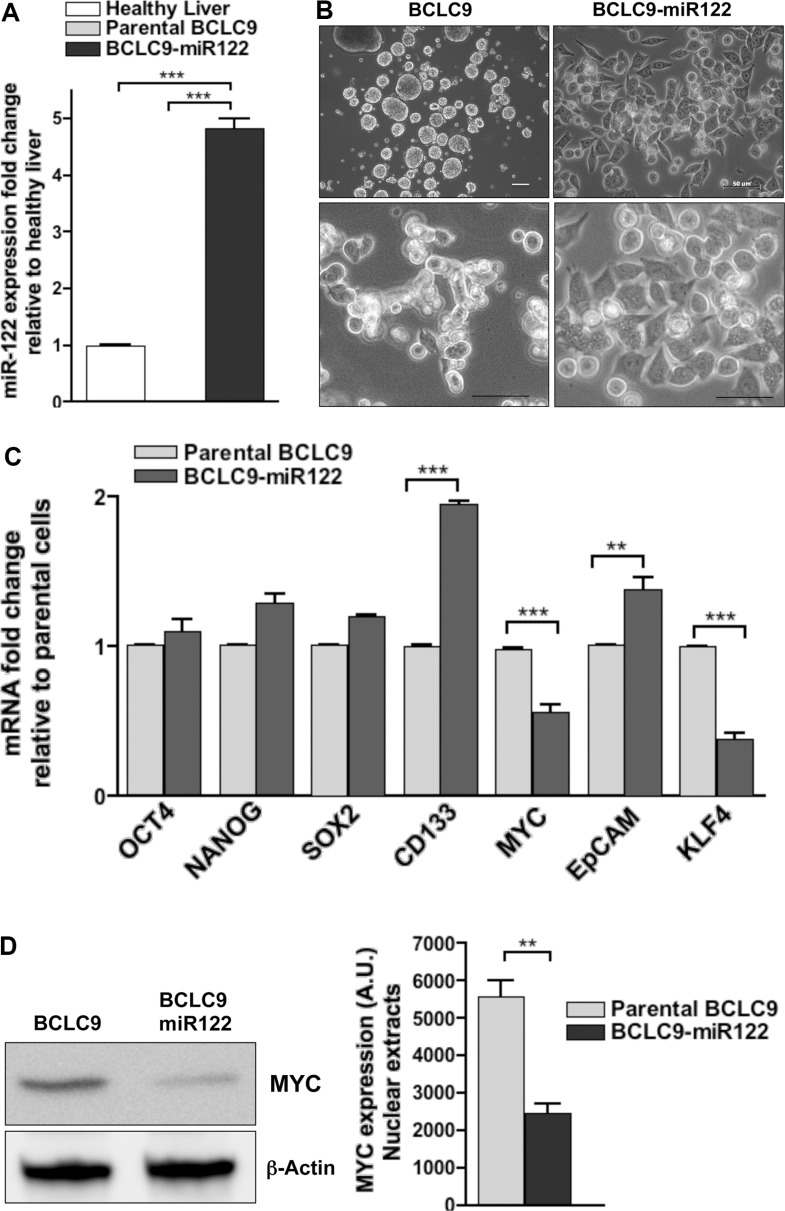
miR122 changes CSC profile and cell adherence capability (**A**) Mature miR122 levels in parental and miR122-transfected BCLC9 cells determined by real-time PCR and related to healthy liver. Results are normalized to *RNU6B* gene. (**B**) Cell adherence in parental and miR122 transfected cells. Scale bars, 50 μm. (**C**) *OCT4, NANOG, SOX2, CD133, MYC, EpCAM,* and *KLF4* gene expression determined by real-time PCR, in BCLC9-miR122 relative to parental cells. Results normalized against *RPLP0* gene. (**D**) IB analysis of MYC in purified nuclear fractions of parental and BCLC9-miR122 cells, β-Actin is loading control.

BCLC9 cell line was established from a well-differentiated human HCC [12] and they show a stem cell phenotype characterized by gene and protein expression of a pool of pluripotency markers: *OCT4, NANOG, SOX2, KLF4, CD133, EpCAM, KRT19* genes, and overexpression of *MYC* which is not due to *MYC* gene amplification (Supplementary Figure S1A–S1C). BCLC9 cells have been authenticated by ATCC as human origin, and not a match for any other profile in the ATCC or DSMZ Short Tandem Repeat (STR) databases. We used Fluorescence *In Situ* Hybridization (FISH) to confirm BCLC9 karyotype previously described for this cell line [12] (Supplementary Figure S2). BCLC9 usual growth pattern is non-adherent spheroid-like structures with a high nucleus to cytoplasmic ratio and they are highly efficient tumor initiating cells in SCID mice. Since BCLC9 cells do not express miR122, they are the perfect setting to analyze the effects of restoring miR122 expression in CSC-like human HCC cells. So, we generated a stable BCLC9 cell line expressing miR122 by plasmid transfection and confirmed its expression by real-time PCR (Figure 1A). BCLC9-miR122 cells show adherent phenotype (Figure 1B) different from that of parental cells. We analyzed the presence of pluripotency cell markers to pinpoint miR122 role in cell differentiation. Only two of the genes tested-*MYC* and *KLF4*-were significantly down-regulated in BCLC9-miR122 cells, while CD133 and EpCAM genes were up-regulated (Figure 1C). We also confirmed a significant decrease in MYC protein load in BCLC9-miR122 cells (Figure 1D). Thus, miR122 might act as a differentiation factor in BCLC9 cells since it down-regulates two out of the four master pluripotency genes: *OCT4, SOX2, KLF4,* and *MYC* [13].

### miR122 reduces cell proliferation and tumor progression *in vivo*

We performed cDNA microarray analysis in three independent batches of both parental BCLC9 cells and BCLC9-miR122 cells grown *in vitro*. Samples were hybridized to Human Genome U219 array plate. A total of 2432 genes that were differentially expressed (1215 genes were up-regulated and 1217 down-regulated) in BCLC9-miR122 compared to BCLC9 cells with a FDR < 0,05 (*p* < 0,05), were used for the analysis using Ingenuity^®^ Pathways Analysis^™^ (IPA) (http://www.ingenuity.com, Ingenuity^®^ Systems, Redwood City, CA, USA). Genes were mapped to genetic networks available in the IPA database and ranked by score. Results of IPA analysis showed a clear enrichment in cell cycle, DNA replication, recombination and repair, and cancer pathways (Supplementary Figure S3A, S3C).

**Figure 2:**
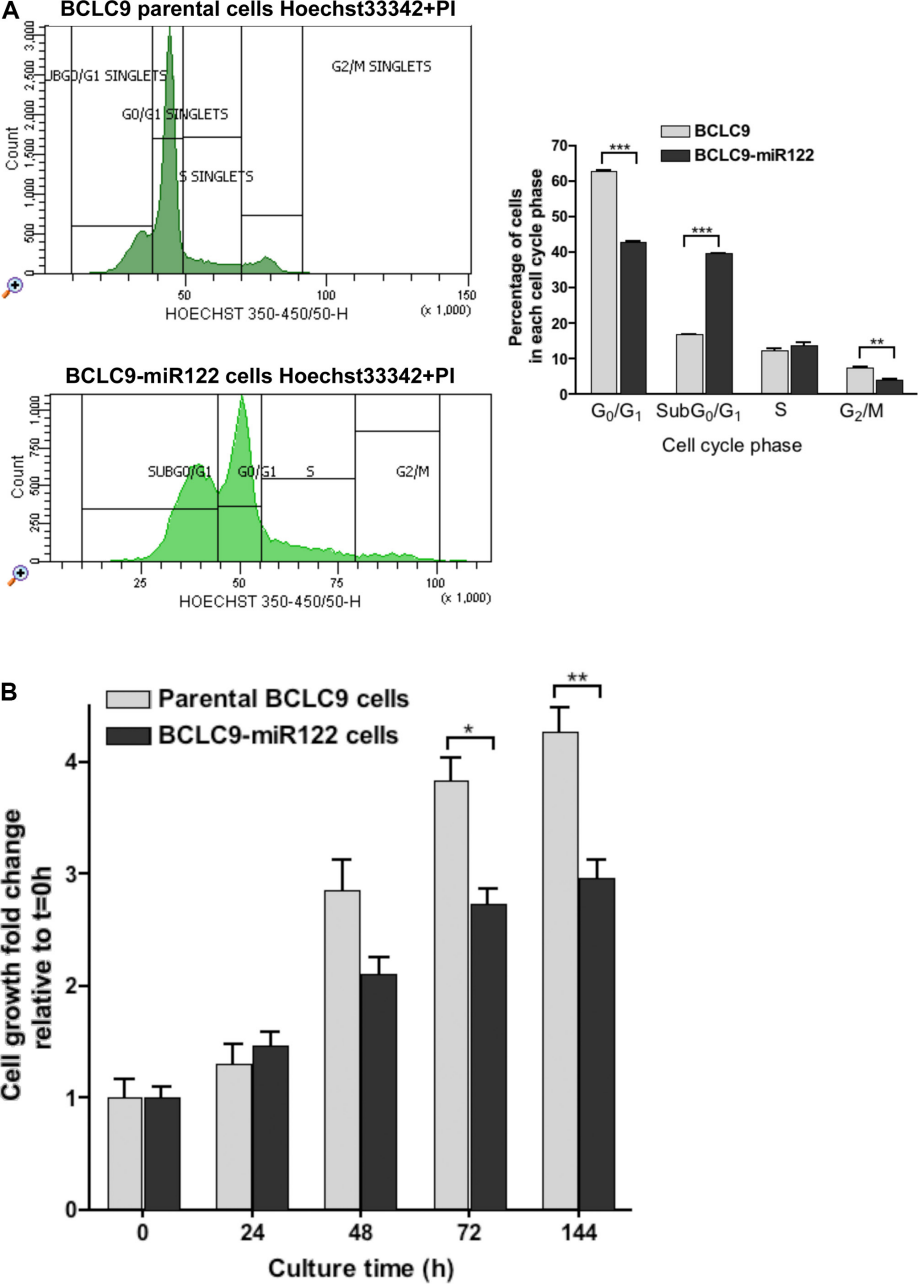

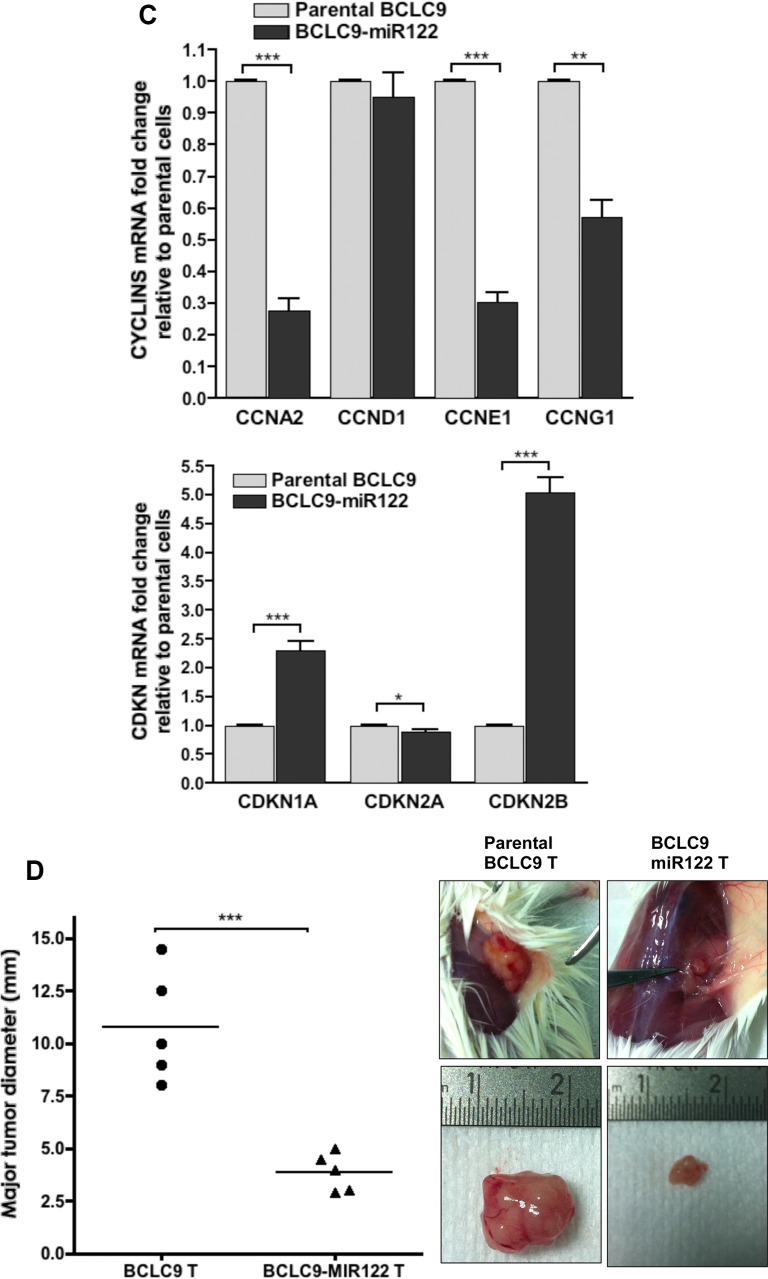
miR122 delays cell proliferation and cell cycle progression (**A**) Flow Cytometry cell cycle analysis in unfixed BCLC9 parental cells (top) and BCLC9-miR122 cells (bottom) in physiologic conditions using Hoechst 33342 and PI as non-vital DNA dye. Graphic shows the percentage of cells in each cell cycle phase. (**B**) Cell growth kinetics at different time points (*t* = 0, 24, 48, 72 and 144 hours) of cell culture. (**C**) *CCNA2, CCND1, CCNE1, CCNG1, CDKN1A, CDKN2A,* and *CDKN2B* gene expression determined by real-time PCR, in BCLC9-miR122 relative to parental cells. Results normalized against *RPLP0* gene. (**D**) Comparison of major tumor diameters (mm) and representative tumors generated by BCLC9 and BCLC9-miR122 cell injection in SCID mice.

We analyzed BCLC9 and BCLC9-miR122 cell cycle by flow cytometry in physiologic conditions, this allowed us to know the percentage of cells alive in each phase. Analysis revealed a high percentage of BCLC9 and BCLC9-miR122 cells in Sub G_0_/G_1_ and G_0_/G_1_ phases (Figure 2A). However, BCLC9-miR122 show a significantly higher Sub G_0_/G_1_ cell population compared to BCLC9. *In vitro* cell proliferation assays along time demonstrate that miR122 reduces significantly cell proliferation rate (Figure 2B). These results are supported by the significant down-regulation of cyclins: *Cyclin A2* (*CCNA2*), *Cyclin E1 (CCNE1*) and *Cyclin G1 (CCNG1*) and up-regulation of *p21* (*CDKN1A*) and *p15 (CDKN2B*) shown by array platform, and confirmed by real-time PCR (Figure 2C). To check miR122 effects on cell proliferation *in vivo*, we injected 1·10^6^ of parental BCLC9 or BCLC9-miR122 cells in SCID mice. Thirty days after injection, mice were sacrificed and tumors were collected and measured, and maximum tumor diameter was registered. All mice from both groups developed tumors, although tumors originated from BCLC9-miR122 cells were significantly smaller than those generated by parental cells (Figure 2D).

Mature miR122 was positively localized in hepatocytes of all tumors from BCLC9-miR122 cells (Supplementary Figure S3B). These results ruled out the possibility that BCLC9-miR122 tumors developed from BCLC9-miR122-negativized cells.

### miR122 triggers dormancy program

**Figure 3:**
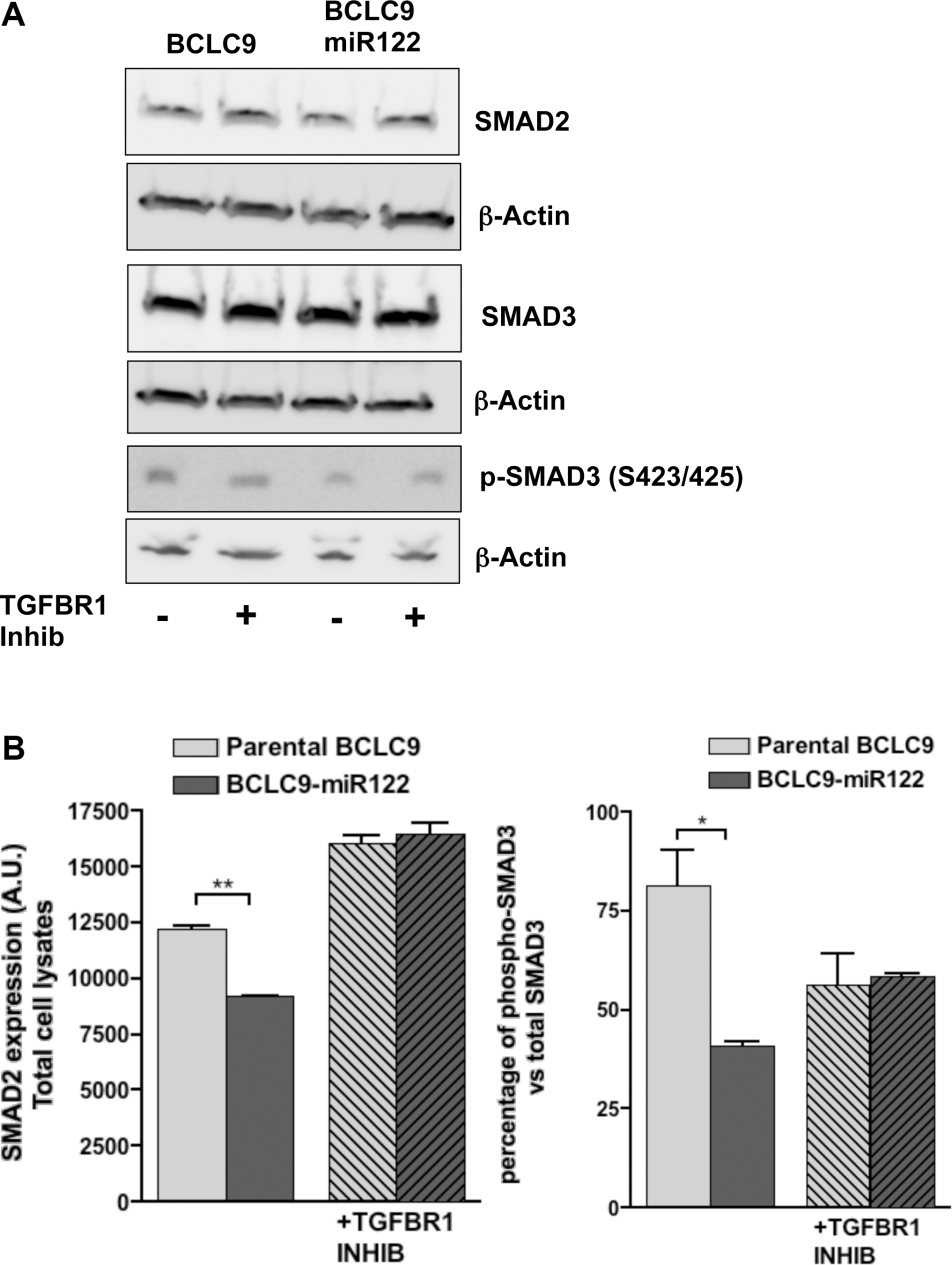

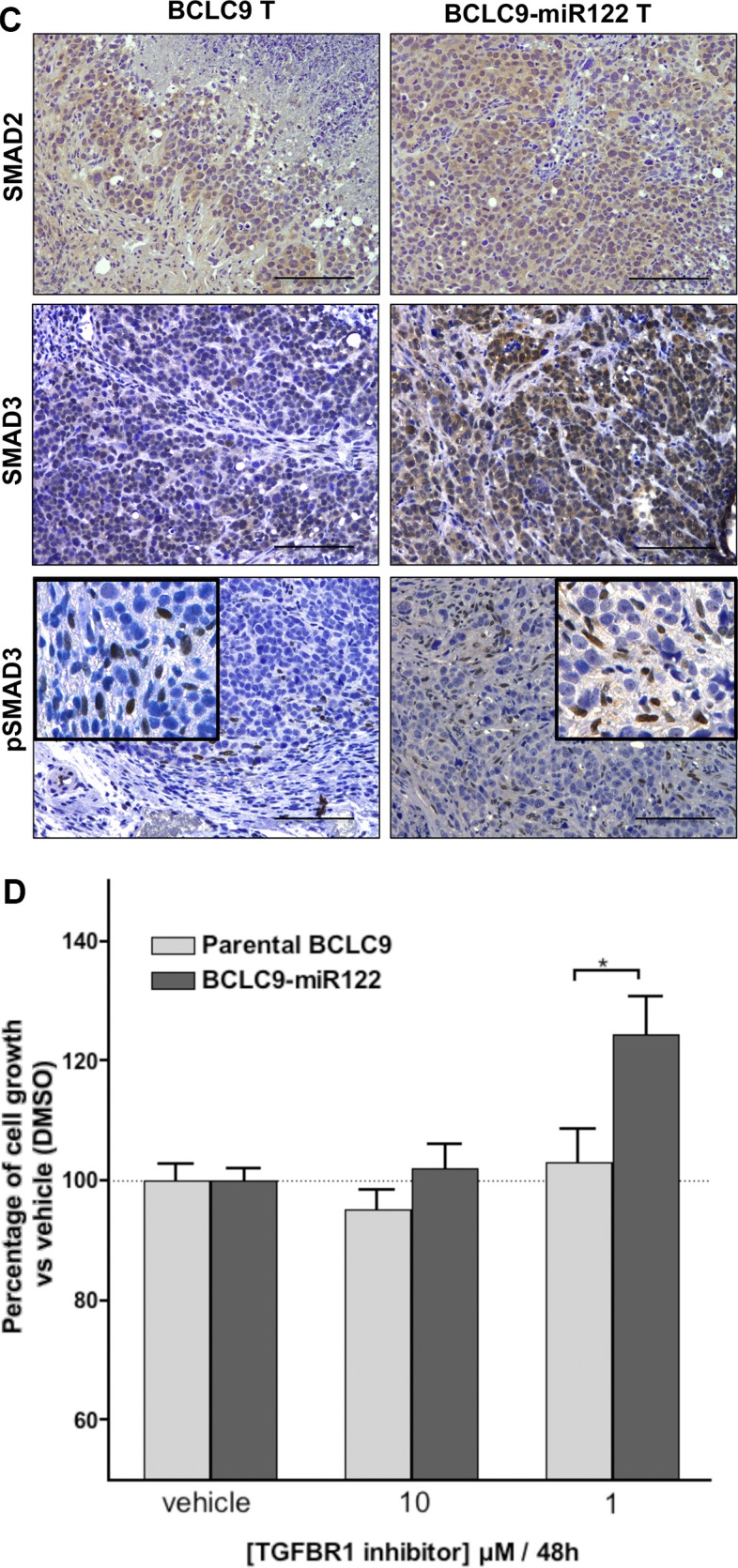
miR122 do not activate canonical TGFβ pathway (**A**) SMAD2, SMAD3, and phospho-SMAD3 IB with (+) and without (−) TGF-β-R1 inhibitor treatment. β-Actin is used as control protein load. (**B**) IB quantification of SMAD2, SMAD3 and phospho-SMAD3. Phosphorylated proteins are expressed as a percentage of their total forms. (**C**) Analysis of SMAD2/SMAD3 expression and phosphorylation in xenograft tumors. Scale bars, 50 μm. p-SMAD3 stainings show a magnification to see cell stroma positive signal. (**D**) TGF-β-R1 inhibitor effects at a concentration of 10 and 1 μM for 48 hours on cell growth kinetics. Treatment is compared to vehicle (DMSO) treated cells.

TGF-β is an anti-mitogenic cytokine that turns into oncogenicity in advanced tumors [14]. We analyzed the potential role of TGF-β pathway in BCLC9-miR122 cells, because the mechanism of TGF-β growth arrest is related to the inhibition of *MYC* expression [15] and the induction of both p21 and p15 genes [16]. Moreover, SMAD4 pathway is listed as an activated pathway in IPA analysis (Supplementary Figure S3C) in BCLC9-miR122 cells. We also confirmed the induction of two TGF-β target genes different from those directly involved in cell cycle progression: TGF-β Induced (*TGFBI*) and TGF-β Receptor 3 (*TGFBR3*) in BCLC9-miR122 cells (Supplementary Figure S3D). Since TGF-β pathway seems to be activated in miR122 transfected cells, we analyzed SMAD2 and SMAD3 status to confirm a potential shift of TGF-β towards a cytostatic role. Neither SMAD2 nor SMAD3 expression and activation, change depending on the presence of miR122 *in vitro* (Figure 3A, 3B) or *in vivo* (Figure 3C). To discard any contribution of TGF-β pathway in BCLC9-miR122 cells, we treated transfected and parental cells with an inhibitor of TGF-β type 1 receptor phosphorylation: TGF-β-R1 kinase inhibitor II (II, 2-(3-(6-Methylpyridin-2-yl)-1H-pyrazol-4-yl)-1,5-naphthyridine). BCLC9-miR122 cells treated with 1 μM of TGF-β-R1 inhibitor for 48 hours significantly increases cell proliferation rate when compared to untreated cells (Figure 3D).

**Figure 4:**
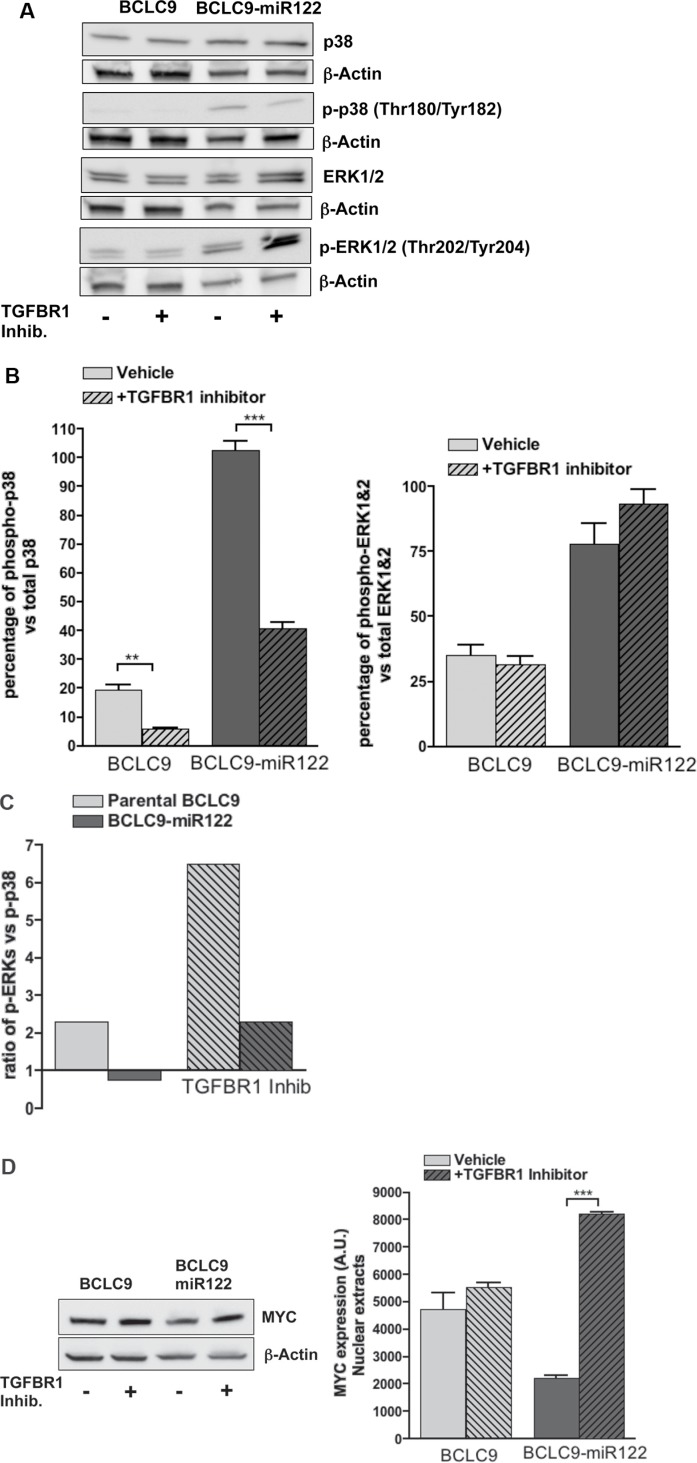
TGF-β-R1 kinase inhibition in BCLC9-miR122 (**A**) IB analysis of total p38, phosphorylated-p38, total ERK1&2, phosphorylated-ERK1&2, with (+) and without (−) TGF-β-R1 inhibitor treatment. β-Actin as control protein load. (**B**) IB quantification of total p38, phosphorylated-p38, total ERK1&2, and phosphorylated-ERK1&2. Phosphorylated kinases are expressed as a percentage of the total protein. (**C**) Ratio phospho-ERK1&2/phospho-p38 with and without TGF-β-R1 inhibitor treatment. (**D**) IB analysis of MYC with (+) and without (−) treatment with TGF-β-R1 inhibitor (1 μM, 48 h). β-Actin as control protein load.

Alternative TGF-β signaling pathways such are activation of ERKs, JNKs and p38 MAP kinases have been described [17]. Besides, recent reports link cancer cell dormancy with a shift in ERK1/2:p38 ratio [18], high p38 over ERK1/2 levels is characteristic of cell dormancy while lower p38 over ERK1/2 is critical for fast growing tumors. To determine if BCLC9-miR122 tumors are going into a dormant state and the potential role of TGFβ pathway in this condition, expression of ERK1/2, and p38 and their phosphorylated forms in parental BCLC9 and BCLC9-miR122 cells were analyzed *in vitro*. We observed a significant increase in the percentage of activated p38 in miR122 transfected cells compared to parental cells (Figure 4A, 4B), inducing a low phosho-ERK1/2:phospho-p38 ratio (Figure 4C). TGF-β-R1 inhibition decreases the percentage of phospho-p38 while further increases phospho-ERK1/2 levels These results confirm that treatment with TGF-β-R1 inhibitor reverts BCLC9-miR122 dormancy towards a proliferative condition due to a significant reduction in p38 activation (Supplementary Figure S4A). Regarding ERK1/2, p38 expression and their activated forms in xenograft tumors, we observed that BCLC9 tumors show widespread cytoplasmic positive ERK1/2 staining and phospho-ERK1/2 nuclear positivity. BCLC9-miR122 tumors show cytoplasmic positive ERK1/2 staining, but no phospho-ERK1/2 (Supplementary Figure S4B). Concerning p38 and phospho-p38 status, BCLC9 tumors have weak cytoplasmic positivity for p38 protein and no nuclear phospho-p38 staining. On the contrary, BCLC9-miR122 tumors show widespread cytoplasmic positivity for p38 and phospho-p38 is clearly localized in the cell nuclei, indicating active p38 protein. Staining pattern in BCLC9-miR122 tumors is comparable to that of mouse healthy liver, used as a reference of quiescent liver. Thus, restoration of miR122 induces a dormant state that can be reverted by TGF-β-R1 pathway inhibition. Furthermore, we analyzed MYC expression during TGF-β-R1 inhibition and we found a significant increase in MYC protein load in BCLC9-miR122 cells (Figure 4D) and a significant decrease in p21 and p15 expression (Supplementary Figure S4C), confirming that TGF-β-R1 signalling pathways exert a cytostatic role in BCLC9-miR122 cells.

### miR122 restores liver cell homeostasis in CSC

**Figure 5:**
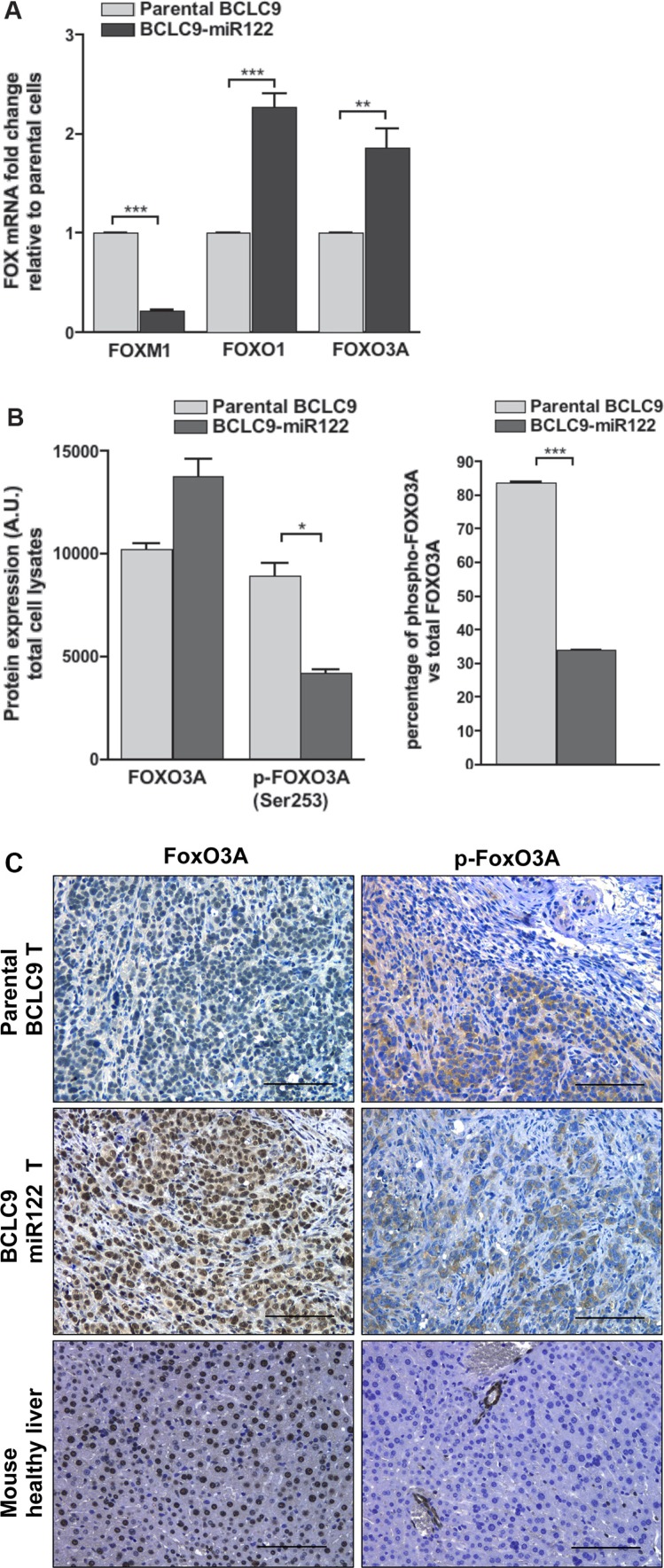
miR122 modulates *FOX* gene family expression (**A**) *FOXM1, FOXO1,* and *FOXO3A* gene expression by real-time PCR. Results are normalized against *RPLP0* gene. (**B**) FOXO3A protein expression and phosphorylation were analyzed by IB. Phosphorylated kinases are expressed as a percentage of the total protein. (**C**) FOXO3A protein expression and phosphorylation analyzed in xenograft tumors by IHC staining. Mouse healthy liver was used as a control for quiescent normal liver. Scale bars, 50 μm.

IPA results showed differential expression of several Forkhead box (FOX) transcription factors, FOXM1, FOXO1 and FOXO3A (Supplementary Figure S3C). *FOXM1* down-regulation was confirmed in BCLC9-miR122 compared to parental cells (Figure 5A). Since, FOXM1 has been associated to human epithelial progenitor cell expansion [19] and malignancy [20], the results support the activity of miR122 in CSC differentiation. FOXO3A functions downstream of several oncogenic pathways [21], including the ERK and PI3K-AKT signalling cascades [22] and it, also, decreases *MYC* expression [23]. We observed *FOXO1* and *FOXO3A* gene up-regulation in BCLC9-miR122 cells in array platform and real-time PCR (Figure 5A). FOXO function is regulated through protein inactivation, consequently we investigated if protein increase is mirrored by active protein. We demonstrated that the percentage of phosphorylated FOXO3A (indicative of protein inactivation) was significantly lower in BCLC9-miR122 cells compared to parental cells (30% vs 80%) (Figure 5B). BCLC9-miR122 tumors show higher amount of nuclear active FOXO3A staining and minor phospho-FOXO3A staining, compared to BCLC9 tumors and comparable to mouse quiescent liver (Figure 5C).

**Figure 6:**
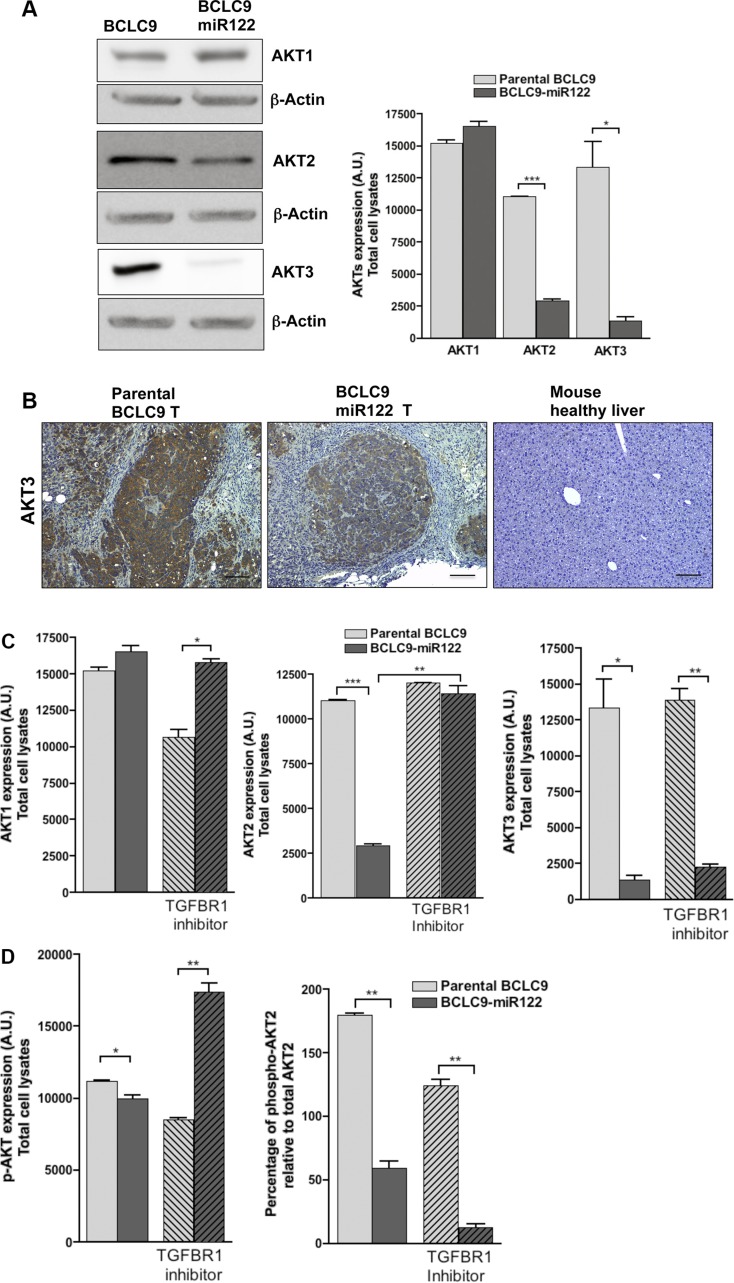

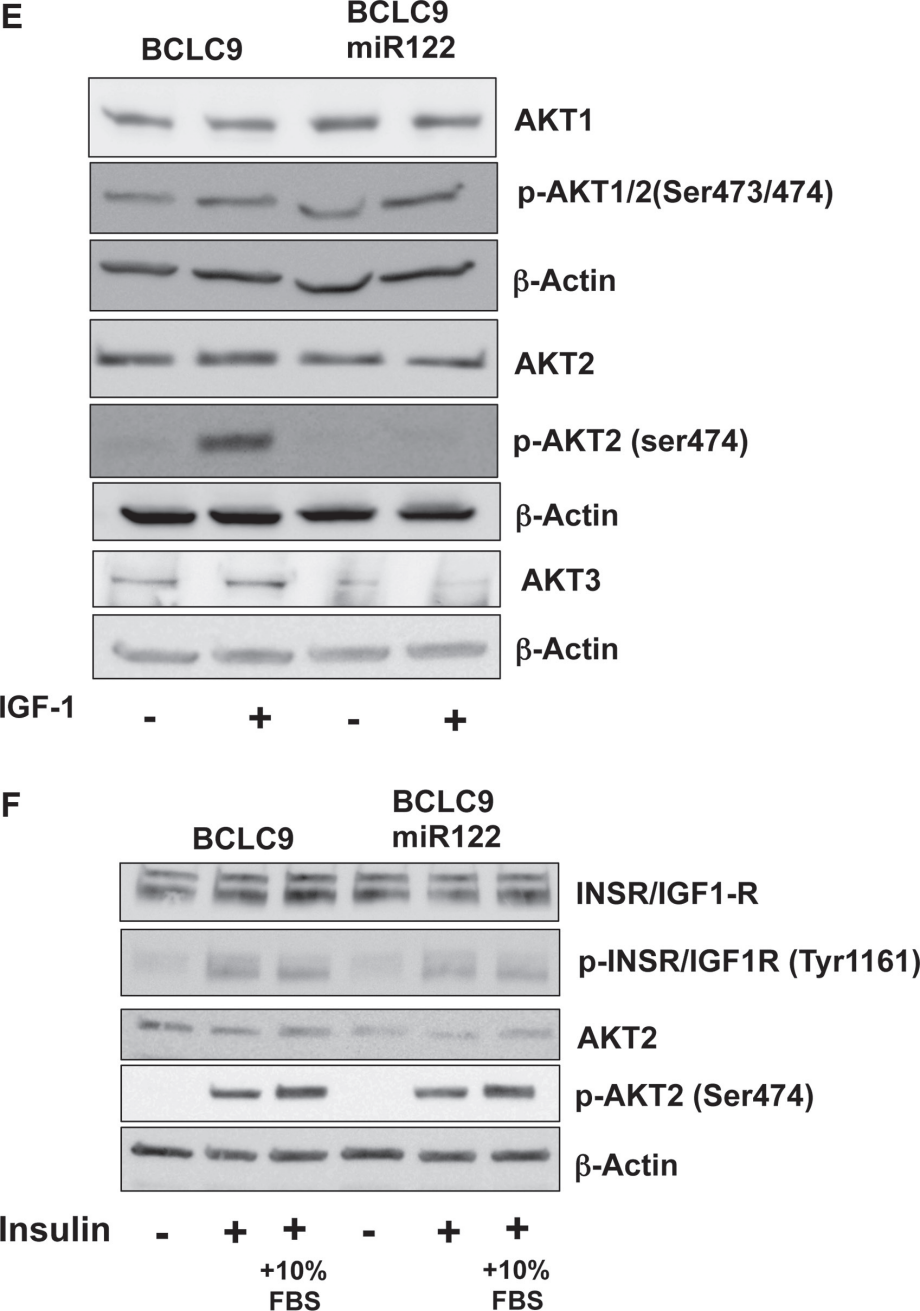
miR122 regulates AKT expression and activation (**A**) IB detection and analysis of AKT1, AKT2, and AKT3 protein expression. β-Actin as control protein load. (**B**) AKT3 detection by IHC in tumors generated by parental and miR122 transfected cells. Mouse healthy liver was used as control of quiescent liver. Scale bars, 50 μm. (**C**) IB quantification of AKT1, AKT2, AKT3 and their phosphorylated forms in parental and miR122 transfected cells with (+) and without (−) TGF-β-R1 inhibitor (1 μM, 48 h). (**D**) Percentage of activated AKT1 and AKT2 isoforms against total load of AKT1 and AKT2 respectively. (**E**) IB detecting AKT1, AKT2, AKT3 and their phosphorylated forms in parental and miR122 transfected cells with (+) and without (−) IGF-1 treatment (10 ng/mL, 30 min.). (**F**) IB detecting AKT1, AKT2, AKT3 and their phosphorylated forms in parental and miR122 transfected cells with (+) and without (−) Insulin treatment (1 μM, 1 h.).

FOXO transcription factors are inactivated thanks to AKT activity. Most cancers exhibit elevated AKT activity due to increased growth factor signaling or oncogenic mutations [24]. Human AKT isoforms (AKT1, AKT2, and AKT3) display non-redundant cellular signaling activities: AKT1 is associated to negative cell cycle progression and proliferation [25], AKT2 contributes to metabolic signaling in the liver [26] and AKT3 is related to brain development [27]. We found a significant down-regulation in all *AKT* gene expression and in AKT2 and AKT3 protein load in miR122 transfected cells (Supplementary Figure S5). AKT3 protein is almost knocked-down in BCLC9-miR122 cells compared to the high amounts of this kinase in parental cells, this ratifies *AKT3* as a miR122 target [28] (Figure 6A). IHC confirmed that AKT3 is highly expressed in BCLC9 tumors, it is reduced in BCLC9-miR122 tumors and completely absent in mouse healthy liver (Figure 6B).

Since TGF-β can also activate PI3K through phosphorylation of its effector AKT [29], we wanted to know if rescue in malignant behavior in BCLC9-miR122 cells due to TGF-β-R1 inhibition, were supported by AKT up-regulated expression and/or activation. TGF-β-R1 inhibition in BCLC9-miR122 cells induced the recovery of AKT2 to the levels observed in parental BCLC9 cells, but AKT3 levels remained nearly undetectable in BCLC9-miR122 (Figure 6C). Percentage of phosphorylated AKT2 over total AKT2 protein load, showed that BCLC9-miR122 cells, with or without TGF-β-R1 inhibitor treatment, do not phosphorylate AKT2, although this treatment induces an increase in AKT2 isoform (Figure 6D). We treated parental and miR122 transfected cells with IGF1, one of the main triggers of AKT activation. AKT2 isoform was phosphorylated after IGF1 treatment in parental BCLC9 cells but not in BCLC9-miR122 cells. AKT1 shows a steady activation through all conditions tested (Figure 6E). To revise the responsiveness of BCLC9-miR122 to IGF1, we used insulin for AKT activation and we found a significant AKT2 phosphorylation in both BCLC9 and BCLC9-miR122 cells (Figure 6F). This result indirectly confirms *IGF1R* as a miR122 target gene [30].

### AKT3 silencing partially reproduces miR122 effects on CSC cells

**Figure 7:**
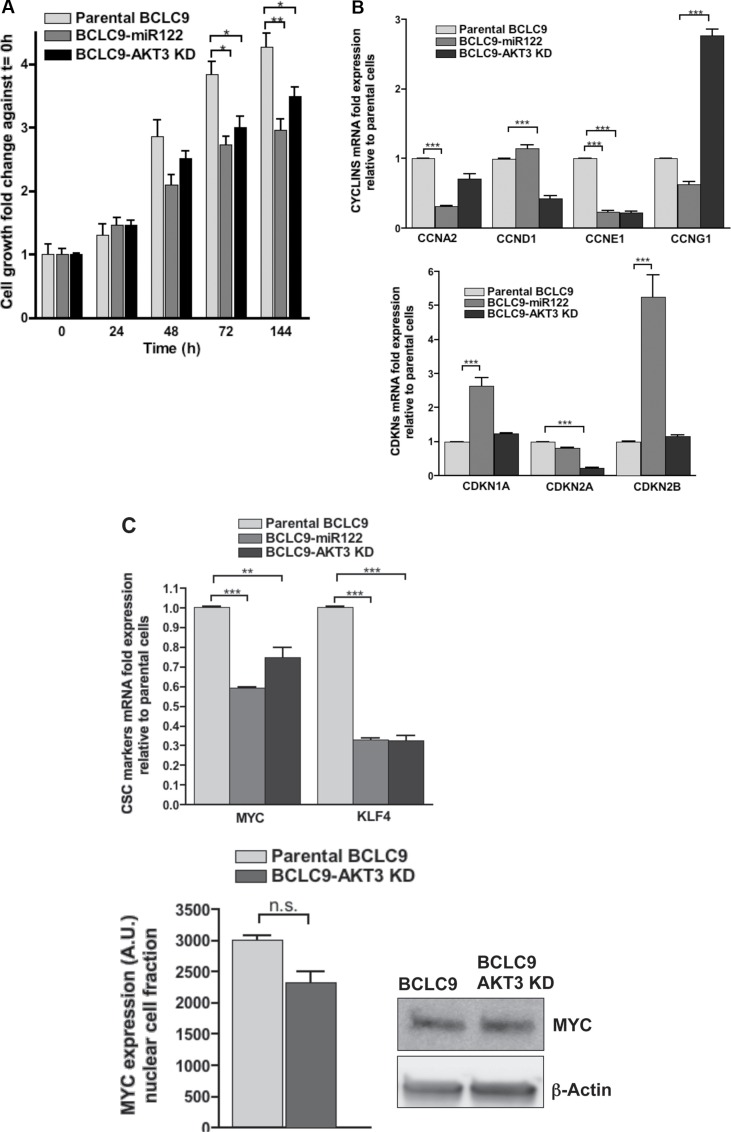

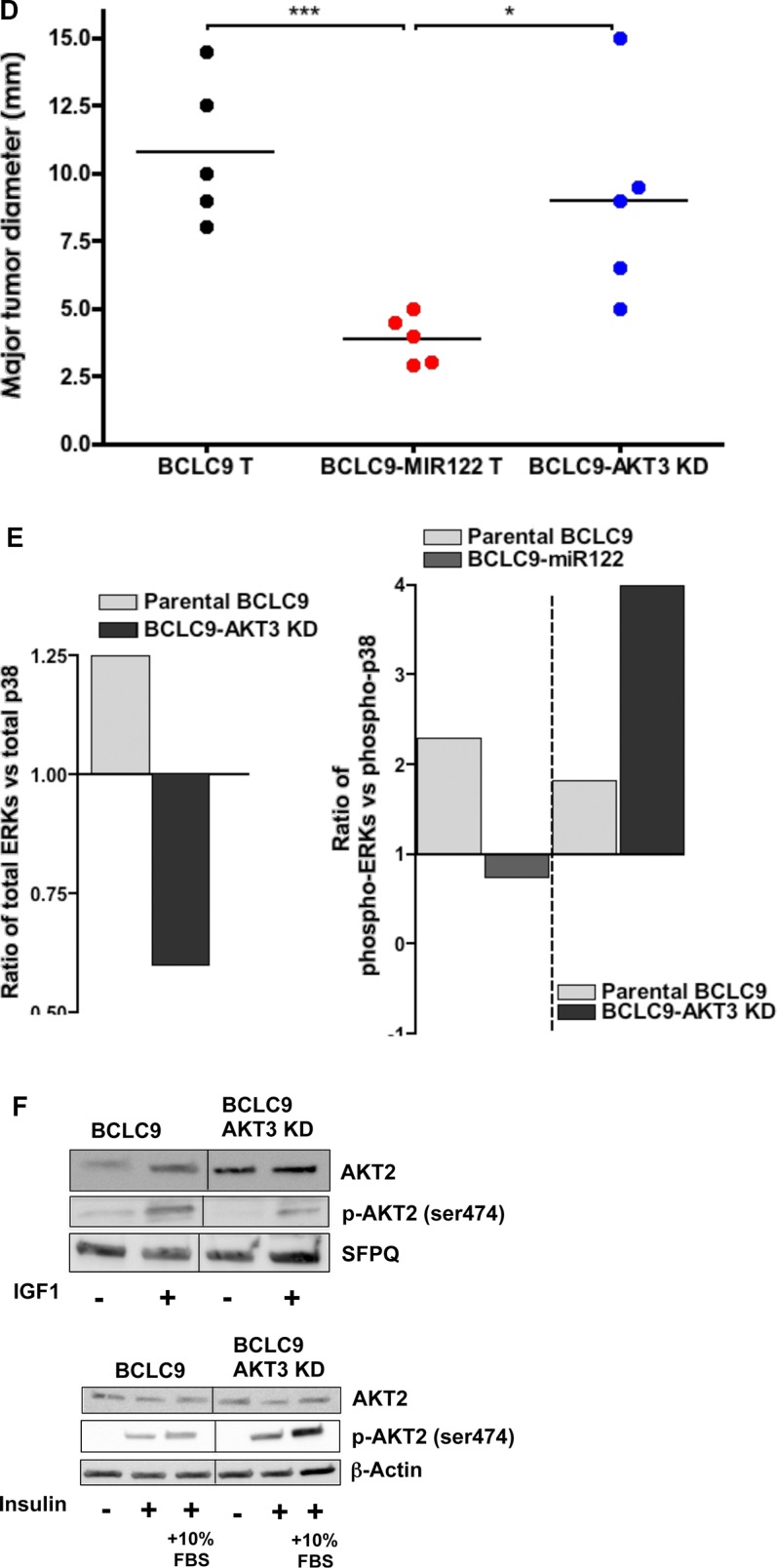
Effects of *AKT3* knock-down (**A**) Cell growth kinetics of parental BCLC9, BCLC9-miR122 and *AKT3* silenced BCLC9 cells at different time points (*t* = 0, 24, 48, 96 and 144 hours). (**B**) *CCNA2, CCND1, CCNE1 CCNG1, CDKN1A, CDKN2A*, and *CDKN2B* gene expression determined by real-time PCR. Results are normalized against *RPLP0* gene. (**C**) *MYC* and *KLF4* gene expression determined by real-time PCR. Results are normalized against *RPLP0* gene. MYC protein detection by IB and quantification. (**D**) Major tumor diameter (mm) obtained in BCLC9, BCLC9-miR122 and BCLC9-AKT3 KD tumors. (**E**) Ratios ERK1&2/p38 in BCLC9-AKT3 KD and parental cells. Ratios of phospho-ERK1&2/phospho-p38 are shown for both BCLC9-miR122 against parental cells and for BCLC9-AKT3 KD against parental cells. (**F**) IB showing the effects of IGF1 (10 ngr/mL, 30 min) and Insulin (1 μM, 1 h) treatments (+) on AKT2 expression and phosphorylation compared to untreated cells (−). Vertical lines are showing the boundaries in vertically sliced gels.

We silenced AKT3 expression in BCLC9 cells to define AKT3 involvement in HCC. We achieved up to a 76% of gene silencing, measured by IB, and confirmed by real-time PCR (Supplementary Figure S6A). AKT3 knocked-down cells (BCLC9-AKT3 KD) show an adherent phenotype comparable to that of BCLC9-miR122 cells (Supplementary Figure S6B) and a lower cell proliferation ratio *in vitro* (Figure 7A). This agrees with a decrease in expression of *CCNA2*, *CCND1* and *CCNE1* in BCLC9-AKT3 KD cells compared to parental and BCLC9-miR122 cells. *CCNG1* increased expression is justified by the fact that cyclin G1 is a miR122 target gene. On the contrary, p21 and p15 expression in BCLC9-AKT3 KD cells is similar to that showed by parental BCLC9 cells (Figure 7B). AKT3 silenced cells showed significantly reduced *MYC* and *KLF4* expression but, we only detected a moderate decrease in MYC protein load (Figure 7C). BCLC9-AKT3 KD cells upregulate *FOXO3A* gene expression similarly as BCLC9-miR122 cells, but *FOXO1* expression do not follow the same trend (Supplementary Figure S6C). *AKT1*, *AKT2* and *AKT3* expression are down-regulated in BCLC9-AKT3 KD cells as observed in BCLC9-miR122 cells (Supplementary Figure S6D). IGF1 and insulin treatments, induced AKT2 phosphorylation in AKT3 silenced cells. Thus, our results demonstrate that miR122 is the unique responsible for rendering BCLC9-miR122 insensitive to IGF1 (Figure 7D).

We analyzed the potential induction of dormancy program in BCLC9-AKT3 KD cells. We observed that, differently from BCLC9-miR122 cells, phospho-ERK1/2:phospho-p38 ratio in BCLC9-AKT3 KD cells was comparable to that in parental BCLC9, clearly favouring phospho-ERK1/2 over phospho-p38 (Figure 7E and Supplementary Figure S6E).

To analyze AKT3-KD cells tumorigenic potential, we reproduced exactly the same protocol previously done in BCLC9-miR122 xenografts. BCLC9-AKT3 KD cells originated tumors which size is halfway between those obtained from parental BCLC9 and BCLC9-miR122 cells (Figure 7F). Although AKT3 absence partially reproduces the effects induced by miR122 expression in BCLC9 cells, it is not enough to fully reduce tumor size. However, they are smaller and hence, still less aggressive than those derived from parental BCLC9 cells.

## DISCUSSION

miR122 is one of the most abundant miRNAs in the adult healthy liver but absent in fetal liver and this is suggestive that miR122 is crucial in organ development. miR122 promotes hepatobiliary segregation and the acquisition and maintenance of a hepato-specific phenotype [8, 31]. Since miR122 is key in hepatocyte differentiation [9], the reported miR122 down-regulation in a high percentage of HCCs, is justified. The existence of liver CSCs is supported by multiple publications [32] and they determine HCC poor prognosis and treatment failure. This backed up the rationale of evaluating the competence of miR122 to revert the cancer stem-like behavior and phenotype exhibited by BCLC9 cells. Multiple sets of cell markers are presumed to be associated to a pluripotent and/or progenitor cell profile [33] in the liver. Available information on this subject, indicates that there is not a sole panel of liver CSC markers but a distinctive CSC behavior, being the main trait the ability in tumor induction [34].

BCLC9 is a human HCC cell line which displays a wide range of CSC markers and has high tumorigenic potential. miR122 transfection in BCLC9 cells is able to reduce significantly the expression of *KLF4* and *MYC* genes, both associated to cell pluripotency. KLF4 is considered to be critical for the maintenance of breast cancer stem cells [35] and to induce cancer development [36]. *MYC*'s central role in cell reprogramming is, also, thoroughly documented [37, 38] as well as its contribution to neoplastic transformation [39, 40]. On the other hand, *CD133* and EpCAM are significantly up-regulated in BCLC9-miR122 cells. CD133 has been considered for a long time a marker for CSCs but there is no consensus on the real contribution of CD133 in the potential of inducing new tumors [41]. On the contrary, EpCAM is a well-established marker for TICs, cancer cells and stem cells [42]. EpCAM contribution to CSC behavior is associated to the induction of *MYC*, *KLF4*, *SOX2*, *NANOG* and *OCT4* expression [43] provided that EpCAM molecule is processed [44]. Therefore, the mere presence of EpCAM does not necessarily imply the acquisition of CSC traits, since EpCAM's activation is mandatory.

Studies performed by Wang and collaborators have shown a potential reciprocal regulation of *MYC* and miR122 in HCC [45]. In agreement with this, we demonstrated that BCLC9-miR122 cells show a lower cell proliferation rate both *in vitro* and in *in vivo* setting. Xenograft tumors derived from BCLC9-miR122 cells are significantly smaller than those generated by parental BCLC9 cells. BCLC9-miR122 reveal a significant decrease in cyclins A2, E1, and G1 and a higher expression of p21, p15 and p16. Not surprisingly, results of array analysis comparing BCLC9-miR122 and BCLC9 cells, revealed an increase in expression of genes involved in cell cycle arrest, and a decrease in DNA replication and cancer pathway-associated genes.

The increase in p21, p15 and p16 expression is related to cell cycle arrest in both quiescence and senescence cell programs [46]. While in cell senescence cell growth-promoting pathways are still active [47, 48], in cell quiescence cell cycle arrest goes with a down-regulation of growth-promoting gene expression [49, 50]. The increase in *p21* and *p15* gene expression [51] and *MYC* gene down-regulation [15] are gene responses associated to TGF-β cytostatic activity. Here we demonstrate TGF-β cytostatic role in BCLC9-miR122 cells by inhibiting TGF-β-R1 kinase activation. After treatment, miR122 transfected cells increased MYC protein load, down-regulated p21 and p15 expression and recovered cell proliferation rate. So, our results support the fact of BCLC9-miR122 cells being in a quiescent state because we are able to reverse this state by inhibiting TGF-β-R1 activation and we are able to demonstrate a down-regulation of IGF1R activity, one of the prevalent growth-promoting genes in malignant cells.

Cancer cell dormancy is defined by a low ERK1/2:p38 ratio [18]. BCLC9-miR122 cells show increased expression of p38 compared to parental cells and the same happens when we determine the ratio of activated forms, phosphoERK1/2:phospho-p38. So, miR122 transfected BCLC9 cells enter in a dormant state and treatment with TGF-β-R1 kinase inhibitor reverts those ratios, thus confirming the exit of dormancy and resuming the high proliferative phenotype in BCLC9-miR122 cells.

IPA analysis unveiled the role of FOX family of transcriptional regulators in BCLC9 cell differentiation. FOXM1 overexpression has been confirmed in a wide range of human cancers and, also, associated to induction of human epithelial progenitor cell expansion [19]. FOXM1 transcription factor coordinates a network of genes that function warranting correct cell cycle progression and exit [52]. We found a significant down-regulation of FOXM1 gene expression in BCLC9-miR122 cells compared to parental BCLC9 cells, thus providing more evidences towards cell differentiation induced by miR122 expression.

FOXO transcription factors are negative regulators of cell proliferation and survival and consequently, they are regarded as tumor suppressors. Thus, loss or inactivation of FOXO proteins, and particularly FOXO3A, will increase resistance to apoptosis and induce cell-cycle progression [53]. This is especially true in tumors since FOXO proteins act downstream of several oncogenic pathways [21] such as ERK or PI3K/AKT [22]. As a matter of fact, both are pathways regulated by non-canonical TGF-β signaling pathway [29]. BCLC9-miR122 cells show a significant increase in *FOXO3A* expression confirmed by an increase in protein load compared to parental cells and a reduced percentage of inactivated FOXO3A. So, miR122 expression is rendering BCLC9 cells to a more “well-behaved” phenotype. Activated AKT stimulate cell growth and proliferation, similarly to the effects observed in response to enhanced expression of MYC [54]. While miR122 restoration induced *AKT2* and *AKT3* significant down-regulation in HCC cells, treatment with TGF-β-R1 kinase inhibitor rescued only *AKT2* expression. Since *AKT3* is a miR122 target, its expression remained almost silenced in miR122 transfected cells whatever treatment we applied. Moreover, BCLC9-miR122 transfected cells only phosphorylate AKT2 kinase under insulin but not IGF1 treatment. This, indirectly confirms *IGF1R* as a gene target of miR122. Since miR122 down-regulation occurs frequently in HCC, upregulation of AKT3 kinase is almost an unavoidable event in this neoplasm.

The role of AKT3 in HCC is not known, so silencing AKT3 isoform in BCLC9 cells determined the effects of miR122 due to AKT3 down-regulation. AKT3 silencing rendered cells more adherent to the culture plate and less proliferative than parental cells, probably due to down-regulation in expression of several cyclins, but not in their corresponding cyclin kinase inhibitors. Since CDKNs are direct targets of TGF-β pathway, our results ratify its cytostatic role in BCLC9-miR122 but not in BCLC9-AKT3 KD cells. Quiescence program was well-defined in BCLC9-miR122 cells, but BCLC9-AKT3 KD cells were not able to activate p38 MAPK in order to keep the low p-ERK1/2:p-p38 ratio that defines dormancy, thus BCLC9-AKT3 KD tumors were bigger than BCLC9-miR122 tumors but smaller than those from BCLC9 cells. AKT3 silenced cells reproduce the expression of *FOXM1* and *FOXO3A* obtained in BCLC9-miR122 cells, but not for *FOXO1* which expression was clearly down-regulated in BCLC9-AKT3 KD cells. In addition, BCLC9-AKT3 KD cells are responsive to IGF1 and insulin treatment, since both induced AKT2 phosphorylation.

Differences between the effects achieved by silencing AKT3 (in a setting of miR122 absence) and miR122 reestablishment, define the key facts in tumor cell dormancy: increase in functional p38 and cell cycle progression inhibitors p21 and p15, modulation of IGF1/IGF1R signaling pathway which, in turn, regulates AKT2 activation and subsequent FOXO activity. In BCLC9 cells, MYC is undoubtedly linked to all pathways regulated by miR122 and *MYC* expression recovery marks the shift in TGF-β activity, from tumor suppressor to oncogene.

Despite the major advances in HCC treatment options [55], therapeutic efficacy is still conditional to underlying liver disease and tumor recurrence. So, it should be key to have a therapy that could work at several fronts.

We reestablished miR122 expression in a distinctive human HCC cell line - the BCLC9 cell line. Its uniqueness relies on the fact that these cells show a complete set of stem/pluripotency stem cell markers, they usually grow forming spheroid structures and they show high efficiency as tumor initiating cells. Moreover, no other cell line displays such stem cell profile.

Our study demonstrates that restitution of miR122 expression, in addition of functioning in stem/progenitor liver cells on a normal basis, is able to reduce tumor growth by inducing tumor cell dormancy in a human HCC cell line with a consistent stem-like profile. Reestablishing miR122 expression should be considered as a potential therapeutic strategy since it would work concurrently reducing tumor aggressiveness and decreasing HCC recurrence by favouring stem-like tumor cell differentiation.

## MATERIALS AND METHODS

### Cell lines

BCLC9 cells were generated as previously described^12^. Standard culture medium for BCLC9 cell lines is Dulbecco's Modified Eagle Medium (DMEM) high glucose (Sigma-Aldrich, St Louis, MO) and HAM F12 (Sigma-Aldrich, St Louis, MO) (1:1). Medium is supplemented with: 1% Na Pyruvate 100 mM (Sigma-Aldrich, St Louis, MO), 1% Pen/Strep 10000 U/mL (Lonza Group Ltd, Switzerland), 1% L-Gln 200 mM (Sigma-Aldrich, St Louis, MO), 1% Non-Essential Aminoacids (NEAA) (Lonza Group Ltd, Switzerland), 10% FBS (Life Technologies). BCLC9-miR122 and BCLC9-AKT3 KD culture medium is supplemented with neomycin (G418 disodium salt, Sigma-Aldrich, Germany; at a final concentration of 500 μgr/mL) and puromycin (Sigma-Aldrich, Germany; at a final concentration of 10 μgr/mL) respectively, once a week to keep selective pressure.

### Cell treatments

1 μM of TGFBR1 inhibitor (Merck #616452) in 1% DMSO for 48 h.

10 ngr/mL IGF-1 (Sigma-Aldrich, St Louis, MO) in 10 nM HCl as vehicle, for 30 minutes.

Human insulin (Sigma-Aldrich, St Louis, MO) 1 μM for 1 h. Lyophilized insulin is resuspended in HEPES buffer and it is diluted in culture medium so control cells were treated with a final concentration of HEPES at 14.5 μM.

Control untreated cells were cultured for the same time period with the corresponding vehicles: 1% DMSO; 10 nM HCl or 14.5 μM HEPES in cell culture medium without FBS.

### Generation of overexpression and knocked-down cells

BCLC9-miR122 cells were obtained by electroporation of pCMV6-GFP-MIR122 plasmid (MI0000442, OriGene Technologies. Rockville, MD). BCLC9-AKT3 KD stable cell line was obtained by lentiviral infection of pGFP-C-shAKT3 (TL320629, OriGene Technologies. Rockville, MD). To obtain stable cell lines, cells were sorted twice by FACS. miR122 and *AKT3* expression was checked by means of real-time PCR using specificTaqMan expression assays (Applied Biosystems, Life Technologies) to detect mature miR122 and *AKT3* expression. Treatment with neomycin once a week keeps selective pressure on miR122-positive cells and puromycin treatment on *AKT3* silenced cells.

### Cell proliferation assay (MTT)

Cell viability and proliferation is determined by 3-(4,5-dimethylthiazol-2-yl)-2,5-diphenyl tetrazolium bromide (MTT) assay (Sigma-Aldrich, St Louis, MO) following manufacturer's instructions. Cells are seeded in 96 well plates and their viability is determined at 0, 24, 48, 72 and 96 hours of cell culture, each time point in triplicates. A byproduct of cell metabolism is measured at 570 nm in a microplate reader (FLUOstar Optima, BMG Labtechnologies Ltd., UK).

### Cell cycle analysis by flow cytometry

Parental BCLC9 and BCLC9-miR122 subconfluent cell cultures are analyzed using Hoechst 33342 (Molecular Probes, Invitrogen) in unfixed cells. Fixation and permeabilization is not needed for labelling cells but physiologic conditions are required because dye internalization is done by the ATP-binding cassette (ABC) transporter activity. Simultaneously, we used Propidium Iodide (PI) (Molecular Probes, Invitrogen) as non-vital DNA dye to discriminate dead cells [56, 57]. Data on DNA content is acquired in a flow cytometer (BD LSRFORTESSA^™^ X-20) and data is analyzed in FACSDiva^™^ Software Version 6.1.3.

### Nucleic acid extraction

MirVana^™^ miRNA isolation Kit (Ambion, Life Technologies) was used to isolate total RNA (including miRNAs) according to manufacturer's protocol. Genomic DNA (gDNA) was purified from cell cultures by means of phenol:chloroform method, following the protocol of Sambrook and Russell [58]. Total gDNA and RNA concentration and purity was measured in a NanoDrop ND-1000 Spectrophotometer (Thermo Scientific, Wilmington DE).

### cDNA microarray analysis

Total RNA was extracted from three independent BCLC9 and BCLC9-miR122 cell batches. RNA samples were further analyzed for its concentration and quality and were hybridized to Human Genome U219 GeneChip from Affymetrix (Affymetrix, Santa Clara, CA) and labeled. The labelled arrays were scanned with a GeneChip scanner 3000 (Affymetrix, Santa Clara, CA). Microarray data were normalized using the guanidine-cytosine content-adjusted robust multiarray algorithm, which computes expression values from probe-intensity values incorporating probe-sequence information.

### Reverse transcription and real-time PCR

Two μgr of total RNA were used for reverse transcription using a High-Capacity cDNA Reverse Transcription Kit (Applied Biosystems, Life Technologies) following the manufacturer's instructions. For multiplexed quantitative real-time PCR, 20 ngr of cDNA template were amplified by means of TaqMan Gene Expression Assay technology (Applied Biosystems, Life Technologies) in an ABI 7500 PCR system. Each target gene analyzed and *huRPLP0* (housekeeping gene) were amplified simultaneously in each well to guarantee the homogeneity in technical replicates. In case of miR amplification, reverse transcription is performed using specific primers followed by monoplexed real-time PCR for target miR and *RNU6B* (housekeeping gene) genes. A complete list of TaqMan gene expression assays used can be found in Supplementary Table S1A.

### Immunoblotting (IB)

For IB analyses, total protein and nuclear extracts were prepared as described previously [59, 60]. Total protein concentration was determined by BCA system (Pierce; Thermo Scientific, Wilmington, DE).

Total protein (50 μgr/lane) or nuclear protein extracts (30 μgr/lane) were separated on SDS-PAGE reducing gels (BioRad, CA). Blots were hybridized with primary antibodies and positive bands were detected by RapidStep enhanced chemiluminescence (ECL) Reagent (Calbiochem, Merck, Germany). IB were quantified using ImageJ 1.46r software (National Institute of Health, USA).

Primary and secondary antibodies used are listed in Supplementary Table S1B.

### Animal experiments

Severe Combined Immuno Deficiency (SCID) mice (CB17/lcr-*Prkdc*^scid^/lcrlcoCrl mouse, were purchased from Charles River (Charles River Lab International Inc. Wilmington, MA) and maintained under pathogen-free conditions in University of Barcelona animal facilities. Procedures involving animals and their care followed institutional guidelines. Four groups of 6–8 week-old SCID mice (*n* = 5/group) were used. Mice were injected subcutaneously (s.c.) in the right flank with 1·10^6^ cells in Phosphate Buffered Saline (PBS). First group mice were injected with 1·10^6^ parental BCLC9 cells, the second group with 1·10^6^ BCLC9-miR122 cells and the third group of mice with 1·10^6^ BCLC9-AKT3 KD cells. The fourth group received vehicle (PBS). Animals were examined three times per week, after 30 days all mice were euthanized by cervical dislocation. Tumors were excised and their maximum and minimum diameters measured. Tumors were divided in two parts, one was snap-frozen for RNA purification and the other half was fixed in formalin and embedded in paraffin for IHC studies.

### Immunocytochemistry (ICC), Immunohistochemistry (IHC) and Immunofluorescence (IF)

IF was performed on cultured cells previously seeded onto poly-lysine treated cover glasses and fixed with cold Methanol:Acetone (1:1). No antigen retrieval was necessary in these samples. IHC was performed on serial 4 μm sections of HCC xenograft samples, previously formalin fixed and paraffin embedded. Antigen retrieval was performed using PT-Link technology (Dako, Denmark). Diamino benzindine (DAB) detection system (EnVision+ System-HRP (DAB), Dako Denmark) was used to detect positive staining for specific primary antibodies. In case of immunofluorescence staining, secondary antibodies conjugated with Alexa-Fluor488 or Alexa-Fluor568 were used (Molecular Probes. Invitrogen) and Hoechst 33342 (Molecular Probes. Invitrogen) for nuclei counterstain. Stainings were analyzed using an Olympus BX51 microscope equipped with DP71 camera (Olympus Europa SE & CO.KG. Germany).

Primary and secondary antibodies used are listed in Supplementary Table S1B.

### Statistical analysis

Data were analyzed using two-tailed Student's *t* test to compare two groups using GaphPad Software Inc, 5.0 (La Jolla, CA, USA). *P* values to assess statistical significance are as follows: *P < 0.05; **P < 0.01; ***P < 0.001. Data for all figures are expressed as the means + SD of, at least, 3 independent experiments. Statistics for microarray analysis is detailed in Supplementary Materials and Methods.

## SUPPLEMENTARY MATERIALS FIGURES AND TABLES



## References

[1] Guo H, Ingolia NT, Weissman JS, Bartel DP (2010). Mammalian microRNAs predominantly act to decrease target mRNA levels. Nature.

[2] Rosenfeld N, Aharonov R, Meiri E, Rosenwald S, Spector Y, Zepeniuk M, Benjamin H, Shabes N, Tabak S, Levy A, Lebanony D, Goren Y, Silberschein E (2008). MicroRNAs accurately identify cancer tissue origin. Nat Biotech.

[3] Lagos-Quintana M, Rauhut R, Yalcin A, Meyer J, Lendeckel W, Tuschi T (2002). Identification of tissue-specific microRNAs from mouse. Curr Biol.

[4] Jopling CL, Yi M, Lancaster AM, Lemon SM, Sarnow P (2005). Modulation of hepatitis C virus RNA abundance by a liver-specific microRNA. Science.

[5] Esau C, Davis S, Murrays SF, Yu XX, Pandey SK, Pear M, Watts L, Booten SL, Graham M, McKay R, Subramaniam A, Propp S, Lollo BA (2006). miR-122 regulation of lipid metabolism revealed by *in vivo* antisense targeting. Cell Metab.

[6] Gramantieri L, Ferracin M, Fornari F, Veronese A, Sabbioni S, Liu CG, Calin GA, Giovannini C, Ferrazzi E, Grazi GL, Croce CM, Bolondi L, Negrini M (2007). Cyclin G1 is a target of miR-122a, a microRNA frequently downregulated in human hepatocellular carcinoma. Cancer Res.

[7] Kutay H, Bai S, Datta J, Motiwala T, Pogribny I, Frankel W, Jacob ST, Ghoshal K (2006). Downregulation of miR-122 in the rodent and human hepatocellular carcinomas. J Cell Biochem.

[8] Coulouarn C, Factor VM, Andersen JB, Durkin ME, Thorgeirsson SS (2009). Loss of miR-122 expression in liver cancer correlates with suppression of the hepatic phenotype and gain of metastatic properties. Oncogene.

[9] Doddapaneni R, Chawla YK, Das A, Kaur J, Ghosh S, Chakraborti A (2013). Overexpression of microRNA-122 enhances *in vitro* hepatic differentiation of fetal liver-derived stem/progenitor cells. J Cell Biochem.

[10] Torre LA, Bray F, Siegel RL, Ferlay J, Lortet-Tieulent J, Jemal A (2015). Global Cancer Statistics, 2012. CA Cancer J Clin.

[11] Forner A, Llovet JM, Bruix J (2012). Hepatocellular carcinoma. Lancet.

[12] Armengol C, Tarafa G, Boix L, Solé M, Queralt R, Costa D, Bachs O, Bruix J, Capellà G (2004). Orthotopic of human hepatocellular carcinoma in mice: Analysis of tumor progression and establishment of the BCLC-9 cell line. Cancer Res.

[13] Takahashi K, Yamanaka S (2006). Induction of pluripotent stem cells from mouse embryonic and adult fibroblast cultures by defined factors. Cell.

[14] Siegel PM, Massagué J (2003). Cytostatic and apoptotic actions of TGF-β in homeostasis and cancer. Nat Rev Cancer.

[15] Chen CR, Kang Y, Massagué J (2001). Defective repression of *c-myc* in breast cancer cells: a loss at the core of transforming growth factor b growth arrest program. Proc. Natl. Acad. Sci. USA.

[16] Amati B (2001). Integrating Myc and TGF-β signalling in cell-cycle control. Nat Cell Biol.

[17] Bhowmick NA, Zent R, Ghiassi M, McDonnell M, Moses HL (2001). Integrin b1 signaling is necessary for transforming growth factor-β activation of p38 MAPK and epithelial plasticity. J Biol Chem.

[18] Aguirre-Ghiso JA (2007). Models, mechanisms and clinical evidence for cancer dormancy. Nat Rev Cancer.

[19] Gemenetzidis E, Elena-Costea D, Parkinson EK, Waseem A, Wan H, Teh MT (2013). Induction of human epithelial stem/progenitor expansion by FOXM1. Cancer Res.

[20] Blanco-Bose WE, Murphy MJ, Ehringer A, Offner S, Dubey S, Huang W (2008). c-Myc and its target FoxM1 are critical downstream effectors of constitutive androstane receptor (CAR) mediated direct liver hyperplasia. Hepatology.

[21] Calnan DR, Brunet A (2008). The FoxO code. Oncogene.

[22] Yang JY, Zong CS, Xia W, Yamaguchi H, Ding Q, Xie X, Lang JY, Lai CC, Chang CJ, Huang WC, Huang H, Kuo HP, Lee DF (2008). ERK promotes tumorigenesis by ihibiting FOXO3A via MDM2-mediated degradation. Nat Cell Biol.

[23] Delpuech O, Griffiths B, East P, Essafi A, Lam EWF, Burgering B, Downward J, Schulze A (2007). Induction of Mxi1-Sra by FOXO3a contributes to repression of Myc-dependent gene expression. Mol Cell Biol.

[24] Engelman JA, Luo J, Cantley LC (2006). The evolution of phosphatidylinositol 3-kinases as regulators of growth and metabolism. Nat Rev Genet.

[25] Heron-Milhavet L, Franckhauser C, Rana V, Berthenet C, Fisher D, Hemmings BA, Fernandez A, Lamb NJ (2006). Only Akt1 is required for proliferation, while Akt2 promotes cell cycle exit through p21 binding. Mol Cell Biol.

[26] Cho H, Thorvaldsen JL, Chu Q, Feng F, Birnbaum MJ (2001). Akt1/PKBalpha is required for normal growth but dispensable for maintenance of glucose homeostasis in mice. J Biol Chem.

[27] Easton RM, Cho H, Roovers K, Shineman DW, Mizrahi M, Forman MS, Lee VM, Szabolcs M, de Jong R, Oltersdorf T, Ludwig T, Efstratiadis A, Birnbaum MJ (2005). Role for Akt3/protein kinase Bgamma in attainment of normal brain size. Mol Cell Biol.

[28] Nassirpour R, Mehta PP, Yin MJ (2013). miR-122 regulates tumorigenesis in hepatocellular carcinoma by targeting AKT3. PLoS One.

[29] Viñals F, Pouysségur J (2001). Transforming growth factor b1 (TGF-b1) promotes endothelial cell survival during *in vitro* angiogenesis via an autocrine mechanism implicating TGF-α signaling. Mol Cell Biol.

[30] Wang B, Wang H, Yang Z (2012). MiR-122 inhibits cell proliferation and tumorigenesis of breast cancer by targeting IGF1R. PLoS One.

[31] Laudadio I, Manfroid I, Achouri Y, Schmidt D, Wilson MD, Cordi S, Thorrez L, Knoops L, Jacquemin P, Schuit F, Pierreux CE, Odom DT, Peers B (2012). A feedback loop between the liver-enriched transcription factor network and miR-122 controls hepatocyte differentiation. Gastroenterology.

[32] Ma S, Chan KW, Hu L, Lee TKW, Wo JYH, Ng IO, Zheng BJ, Guan XY (2007). Identification and characterization of tumorigenic liver cancer stem/progenitor cells. Gastroenterology.

[33] Kim H, Park YN (2014). Hepatocellular carcinomas expressing “stemness”-related markers: Clinicopathological characteristics. Dig Dis.

[34] Yamashita T, Ji J, Budhu A, Forgues M, Yang W, Wang HY, Jia H, Ye Q, Qin LX, Wauthier E, Reid LM, Minato H, Honda M (2009). EpCAM-positive hepatocellular carcinoma cells are tumor-initiating cells with stem/progenitor cell features. Gastroeneterology.

[35] Yu F, Li J, Chen H, Swapan R, Huang S, Zheng H, Ai W (2011). Krüppel-like factor 4 (KLF4) is required for maintenance of breast cancer stem cells and for cell migration and invasion. Oncogene.

[36] Rowland BD, Peeper DS (2006). KLF4, p21 and context-dependent opposing forces in cancer. Nat Rev Cancer.

[37] Takahashi K, Tanabe K, Ohnuki M, Narita M, Ichisaka T, Tomoda K, Yamanaka S (2007). Induction of pluripotent stem cells from adult human fibroblasts by defined factors. Cell.

[38] Takayama N, Nishimura S, Nakamura S, Shimizu T, Ohnishi R, Endo H, Yamaguchi T, Otsu M, Nishimura K, Nakanishi M, Sawaguchi A, Nagai R, Takahashi K (2010). Transient activation of c-MYC expression is critical for efficient platelet generation from human induced pluripotent stem cells. J Exp Med.

[39] Gabay M, Li Y, Felsher DW (2014). MYC activation is a hallmark of cancer initiation and maintenance. Cold Spring Harb Perspect Med.

[40] Dang CV (2012). MYC on the path to cancer. Cell.

[41] Irollo E, Pirozzi G (2013). CD133: to be or not to be, is this the real question?. Am J Transl Res.

[42] Visvader JE, Lindeman GJ (2008). Cancer stem cells in solid tumours: accumulating evidence and unresolved questions. Nat Rev Cancer.

[43] Lu TY, Lu RM, Liao MY, Yu J, Chung CH, Kao CF, Wu HC (2010). Epithelial Cell Adhesion Molecule regulation is associated with the maintenance of the undifferentiated phenotype of human embryonic stem cells. J Biol Chem.

[44] Denzel S, Maetzel D, Mack B, Eggert C, Bärr, Gires O (2009). Initial activation of EpCAM cleavage via cell-to-cell contact. BMC Cancer.

[45] Wang B, Hsu SH, Wang X, Kutay H, Bid HK, Yu J, Ganju RK, Jacob ST, Yuneva M, Ghoshal K (2014). Reciprocal regulation of microRNA-122 and c-Myc in hepatocellular cancer: Role of E2F1 and Transcription Factor Dimerization Partner 2. Hepatology.

[46] Blagosklonny MV (2014). Geroconversion: irreversible steo to cellular senescence. Cell Cycle.

[47] Leontieva OV, Lenzo F, Demidenko ZN, Blagosklonny MV (2012). Hyper-mitogenic drive coexists with mitotic incompetence in senescent cells. Cell Cycle.

[48] Demidenko ZN, Blagosklonny MV (2008). Growth stimulation leads to cellular senescence when the cell cycle is blocked. Cell Cycle.

[49] Blagosklonny MV (2008). Aging: ROS or TOR. Cell Cycle.

[50] Blagosklonny MV (2012). Tumor suppression by p53 without apoptosis and senescence: conundrum or rapalog-like gerosuppression?. Aging (Albany NY).

[51] Reynisdottir I, Polyak K, Iavarone A, Massagué J (1995). Kip/Cip and Ink4 Cdk inhibitors cooperate to induce cell cycle arrest in response to TGF-β. Genes Dev.

[52] Wierstra I, Alves J (2007). FOXM1, a typical proliferation-associated transcription factor. Biol Chem.

[53] Kops GJ, Medema RH, Glassford J, Essers MA, Dijkers PF, Coffer PJ, Lam EW, Burgering BM (2002). Control of cell cycle exit and entry by protein kinase B-regulated forkhead transcription factors. Mol Cell Biol.

[54] Vanhaesebroeck B, Leevers SJ, Ahmadi K, Timms J, Katso R, Driscoll PC, Woscholski R, Parker PJ, Waterfield MD (2001). Synthesis and function of 3-phosphorylated inositol lipids. Annu Rev Biochem.

[55] Forner A, Gilabert M, Bruix J, Raoul JL (2014). Treatment of intermediate-stage HCC. Nat Rev Clin Oncol.

[56] Belloc F, Dumain P, Boisseau MR, Jalloustre C, Reiffers J, Bernard P, Lacombe F (1994). A flow cytometric method using Hoechst 33342 and Propidium Iodide for symultaneous cell cycle analysis and apoptosis determination in unfixed cells. Cytometry.

[57] Choi T Cell cycle analysis using Hoechst 33342 in unfixed cells. Protocol from Analytical Cytometry/Image Analysis Core Facility; EOHSI.

[58] Sambrook J, Russell DW (2006). Purification of nucleic acids by extraction with phenol:chloroform. Cold Spring Harbor Protocols.

[59] Holden P, Horton WA (2009). Crude subcellular fractionation of cultured mammalian cell lines. BMC Res Notes.

[60] Hattori M, Tugores A, Veloz L, Karin M, Brenner A (1990). A simplified method for the preparation of transcriptionally active liver nuclear extracts. DNA and Cell Biol.

