# Supplementary material for: Restoring miR122 in human stem-like hepatocarcinoma cells, prompts tumor dormancy through Smad-independent TGF-β pathway
Source: Oncotarget. 2016 Sep 7;7(44):71309–29. doi: 10.18632/oncotarget.11885 (PMC5342080; doi:10.18632/oncotarget.11885)
Supplement: Supplementary file 1 [file oncotarget-07-71309-s001.pdf]

Restoring miR122 in human stem-like hepatocarcinoma cells, prompts tumor dormancy through Smad-independent TGF-β pathway

Supplementary Materials

SUPPLEMENTARY EXPERIMENTAL PROCEDURES

Fluorescence *in situ* hybridisation (FISH) of metaphases from BCLC9 cells

In order to check that BCLC9 cell karyotype is maintained along the passages, we performed the simultaneous analysis of all 24 chromosomes using the Chromoprobe Multiprobe System OctoChrome (Cytocell. Cambridge, Engl). Metaphases from BCLC9 were obtained following conventional cytogenetic protocols and were checked for chromosomal amplifications and rearrangements. This system uses an 8 square mutliprobe device and whole chromosome painting probes, labeled in 3 different colour fluorophores (Texas Red, FITC, and Coumarin), each square contains probes for three different chromosomes. This system allows the simultaneous analysis of all 24 chromosomes. We confirmed the previously published chromosome alterations profile (trisomy of chromosome 7, gain of short arm of chromosome 5, partial gain of distal region of the long arm of chromosome 9 that links to chromosome 13).

Gene copy number assay

Twenty nanograms of gDNA are used for copy number analysis in a duplex real-time polymerase chain reaction (PCR) system (Applied Biosystems, Life Technologies). The copy number assay detects the target gene and it is compared to a reference sample that is known to exist in two copies in a diploid genome (RNase P H1 RNA gene).

The number of copies of the target sequence is determined by relative quantitation using the comparative CT (ΔΔCT) method. The copy number of the target is calculated to be two times the relative quantity.

In Situ Hybridization (ISH)

ISH was performed following the manufacturer's instructions (miRCURY LNA™ microRNA detection kit. Exiqon Inc). Briefly, slides were deparaffinized and rehydrated at room temperature (RT). Immediately after, samples were incubated with a 30 µgr/mL Proteinase-K solution for 30 minutes at 37ºC. Slides were rinsed with PBS and directly dehydrated into a series of ethanol solutions (70%, 96%, and 99,9%) and air-dried.

Hybridization mix containing the specific probe (miR-122 probe or U6 probe as a positive control) was applied to each section and incubated for 1 hour at 56ºC. Samples were washed in a series of SSC buffer decreasing concentrations (starting at 5X SSC to 0.2X SSC) at 56ºC. Finally, samples were rinsed with PBS at RT. Slides were incubated with blocking solution for 15 min at RT in a humidifying chamber. Blocking solution was removed and DIG-reagent was applied for 60 minutes at RT. Slides were washed 3 times in PBS-T and incubated with freshly prepared AP substrate for 2 hours at 30ºC. To stop the reaction slides were incubated with KTBT buffer. Slides were rinsed with water and counterstained with Fast Red™ next; samples were mounted. Precipitates must settle down, at least, overnight.

Stainings were analyzed using an Olympus BX51 microscope equipped with DP71 camera (Olympus Europa SE & CO.KG. Germany).

Statistical analysis

Microarray analysis

All statistical analyses were conducted using the R packages "Statistics for Microarray Analysis" (Dudoit et al., 2002). For cDNA microarray analysis all spots from each microarray were included in the analysis. Data normalization was performed using scaled loess normalization and differential genes were identified using an empirical Bayes method for analysing replicated microarray data (Efron et al,. 2001).

The following cutoffs were used in this study: fold change > 2 or < –2 and *P*-value with FDR< 0.05 (Benjamini et al,. 1995).

To define the signaling pathways in which are involved the differentially expressed genes, we used Ingenuity® Pathways Analysis™ (IPA) (http://www.ingenuity.com, Ingenuity® Systems, Redwood City, CA, USA).

The paired two-tailed Student's *t* test was used when comparing two groups. A *P* value less than 0.05 was considered statistically significant.

REFERENCES

1. Benjamini Y, Hochberg Y. Controlling the false discovery rate: a practical and powerful approach to multiple testing. J Royal Statistical Society. 1995; 57:289–300.
2. Dudoit S, Yang YH, Callow MJ, Speed TP. Statistical methods for identifying differentially expressed genes in replicated cDNA microarray expreriments. Statist Sinica 2002; 12:111–139.
3. Efron B, Tibshirani R, Storey JD, Tusher V. Empirical Bayes analysis of a microarray experiment. J American Statistical Association. 2001; 96:1152–1160.

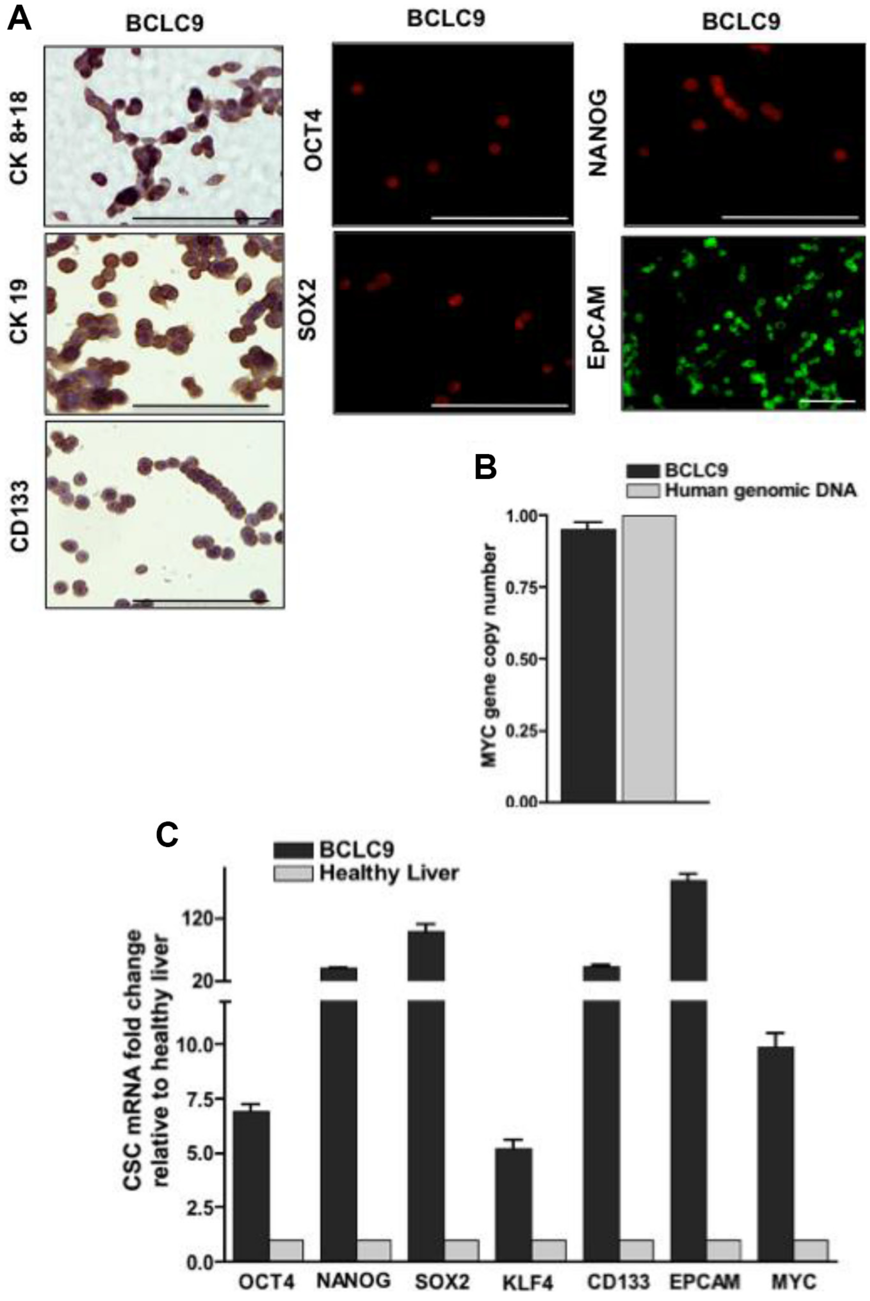

Supplementary Figure S1: BCLC9 cell characterization. (**A**) Immunocytochemistry analysis shows the presence of stem cell markers in BCLC9 cells. Scale bars, 100 µm. (**B**) *MYC* gene copy number assay in BCLC9 cells. Results are normalized against RNase P H1 RNA gene. (**C**) Quantification of CSC markers in BCLC9 cells relative to human healthy liver. Results are normalized against *RPLP0* gene.

A

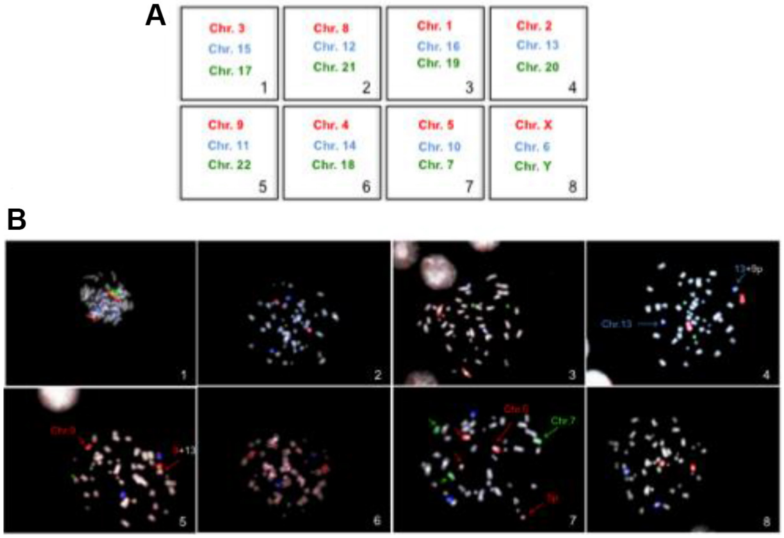

Supplementary Figure S2: BCLC9 cell karyotype. (**A**) Key to identify chromosome-specific probes immobilized in each spot. (**B**) Representative results for metaphase staining by FISH, obtained in the 8 spots.

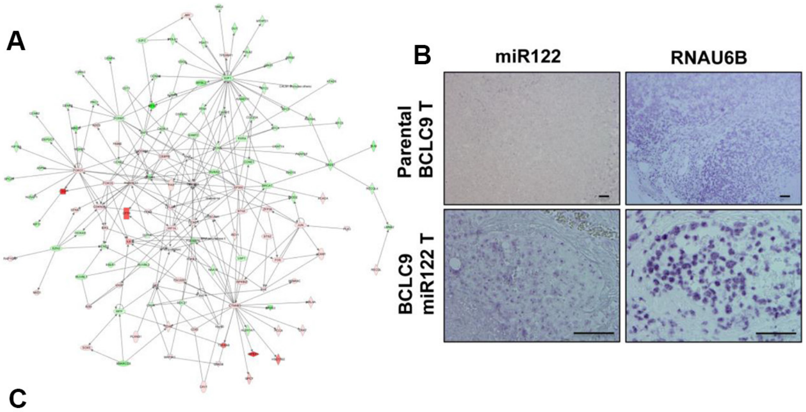

C

Upstream Regulator	Fold Change	Molecule Type	Predicted Activation State	Activation z-score	p-value of overlap	TARGET MOLECULES DATASET
FOXM1	-1.511	transcription regulator	Inhibited	-3.796	6.51E-09	AURKB,BIRC5,BUB1B,CCNA2,CCNB1,CCNE2,CDC20,CDKN1A,CDKN3,CENPA,CENPF,GTSE1,PL
KDM5B		transcription regulator	Activated	3.152	3.36E-08	AURKA,BRCA1,BUB1B,CAV1,CCNB1,CDCA3,DLGAP5,E2F1,GADD45A,HMMR,HSD17B8,KIF2C, MCM2,MIA3,MPC1,MT1X,NDC80,NEDD9,NMB,OIP5,PBK,PDE3B,PIR,POLB,PRPS1,PSD3,PSIP1,RE
GLI1		transcription regulator	Activated	2.020	8.43E-04	CCND1,CDKN1A,FOXA2,ID2,IDS,IL1R2,KRT15,RXRA,TNC,TUFM
ATF3		transcription regulator	Activated	2.000	3.18E-03	AURKA,AURKB,NEK2,PLK1
YAP1	1.844	transcription regulator	Activated	2.000	4.84E-03	CTGF,CYR61,DIAPH3,EDN1,PPP1R3B
FOXO3	2.202	transcription regulator	Activated	2.641	9.99E-03	CCND2,CDKN1A,CDKN2B,CTGF,FOXO1,GADD45A,IER3,IL8
MYB		transcription regulator	Inhibited	-2.200	2.48E-02	CCNB1,CXCR4,DHRS2,PCNA,POLA1
SP1		transcription regulator	Activated	2.000	3.21E-02	ABHD2,CAV1,CCND1,CDKN1A,CDKN2B,CDKN2D,CEACAM1,EIF2AK2,HPSE,IL8,INSR,ITGA2,PR OCR,SLC31A1,TRIB1
FOXO4		transcription regulator	Activated	2.232	3.34E-02	CDKN1A,CDKN2B,CTGF,GADD45A,IER3
ERG		transcription regulator	Activated	2.357	4.35E-02	CCNA2,CD59,CDKN2D,CXCR4,DIAPH3,DNMBP,DOCK1,EXT1,GAK,HMMR,LAMA5,MET,MYO10 ,PHACTR2,PXN,RAPGEF5,RASSF1,RGS3,ROCK2,RRAS,SVIL,TRIOBP,UTRN
SMAD4		transcription regulator	Activated	2.236	4.36E-02	CDKN1A,CDKN2B,CTGF,GADD45A,IER3

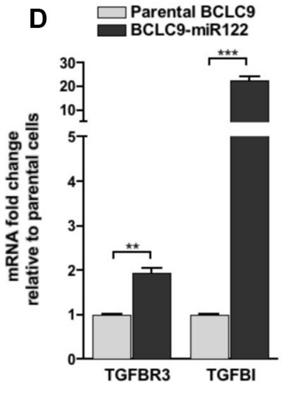

Supplementary Figure S3: Differential gene expression induced by miR122 in BCLC9 cells. (**A**) Network of genes differentially expressed in BCLC9-miR122 cells against parental BCLC9 cells. (**B**) miR122 localization in xenograft tumors by *In Situ* Hybridization. Scale bars, 50 µm. (**C**) Activated and inactivated upstream regulators table obtained in IPA analysis. (**D**) *TGFBI* and *TGFBR3* gene expression analyzed by real-time PCR in parental and miR122 transfected BCLC9 cells. Results are normalized against *RPLP0* gene.

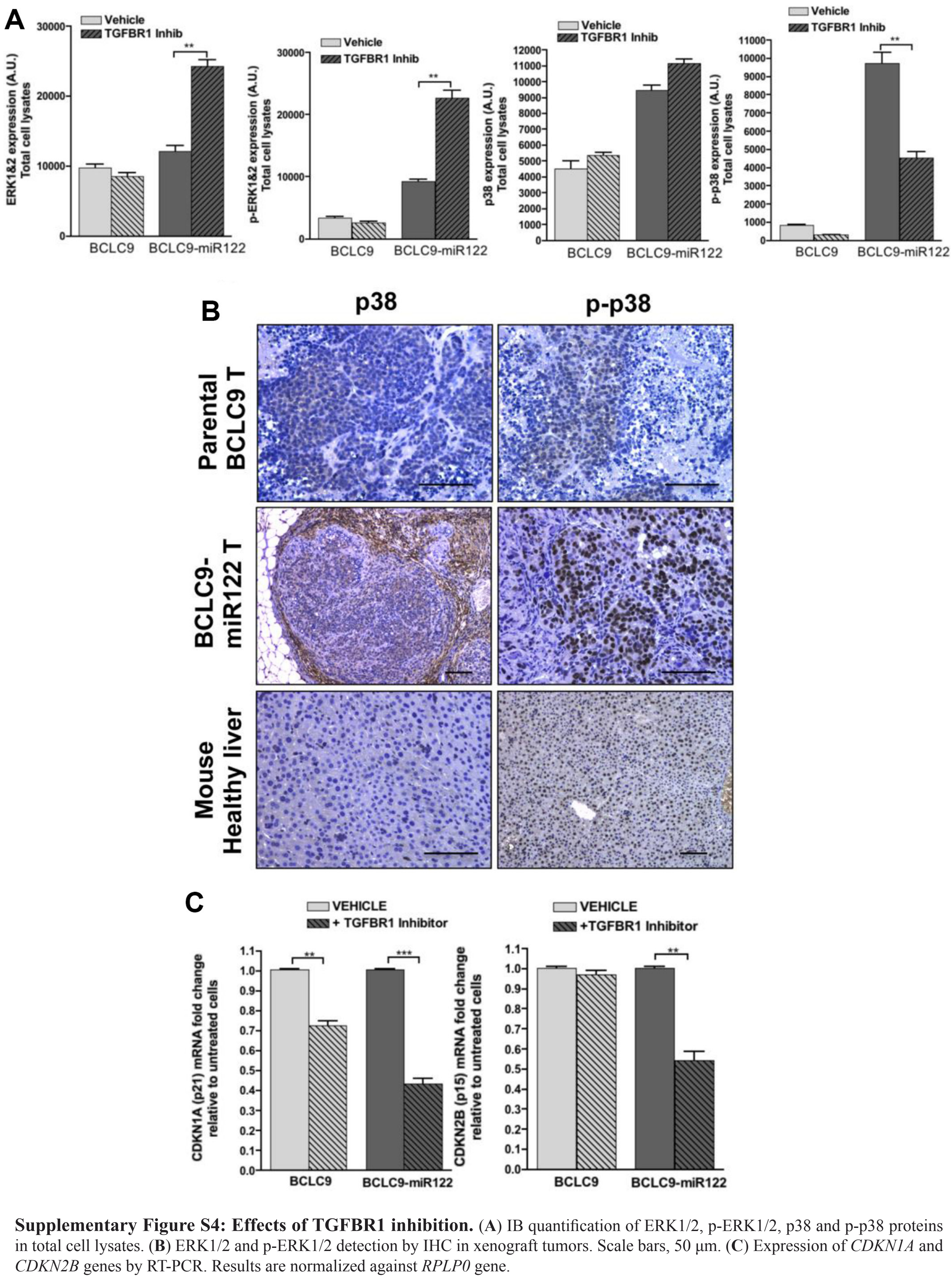

Supplementary Figure S4: Effects of TGFBR1 inhibition. (**A**) IB quantification of ERK1/2, p-ERK1/2, p38 and p-p38 proteins in total cell lysates. (**B**) ERK1/2 and p-ERK1/2 detection by IHC in xenograft tumors. Scale bars, 50 µm. (**C**) Expression of *CDKN1A* and *CDKN2B* genes by RT-PCR. Results are normalized against *RPLP0* gene.

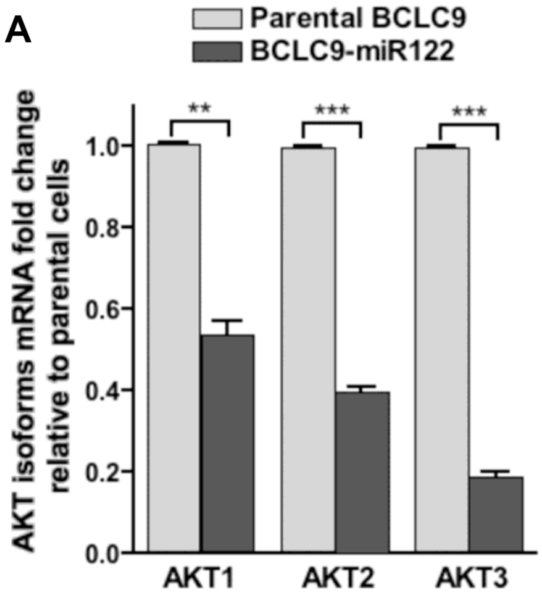

Supplementary Figure S5: *AKT1*, *AKT2* and *AKT3* gene expression in parental BCLC9 and BCLC9-miR122 cells. *AKT1, AKT2,* and *AKT3* gene expression was determined by real-time PCR. Results are normalized against *RPLP0* gene.

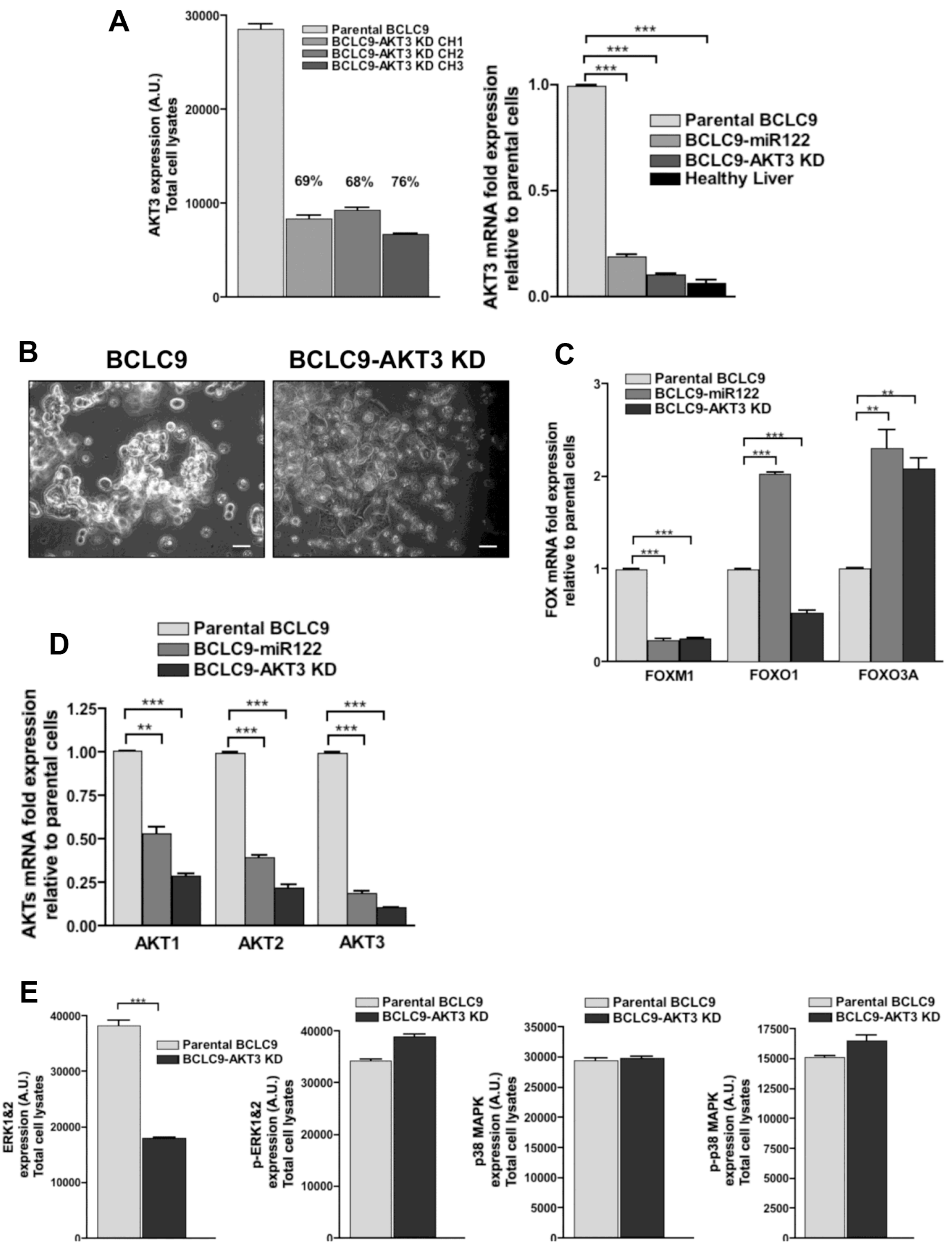

Supplementary Figure S6: Effects of AKT3 silencing in BCLC9 cells. (**A**) IB representing the percentage of AKT3 silencing in three different BCLC9 clones. *AKT3* gene expression determined by real-time PCR in parental BCLC9, BCLC9-miR122, BCLC9 AKT3 KD and healthy liver. Expression is normalized against *RPLP0* gene. (**B**) Change in BCLC9 cell adherence due to *AKT3* silencing. Scale bars, 50 µm. (**C**) *FOXM1*, *FOXO1* and *FOXO3A* gene expression determined by real-time PCR in parental BCLC9, BCLC9-miR122 and BCLC9-AKT3 KD cells. Expression is normalized against *RPLP0* gene. (**D**) *AKT* isoforms expression by Real-Time PCR. Results are normalized against *RPLP0* gene. (**E**) IB quantification of ERK1/2, p-ERK1/2, p38 and p-p38 proteins in total cell lysates in BCLC9-AKT3 KD and parental BCLC9 cells.

Supplementary Table S1A: list of taqman gene expression assays

Gene	TaqMan gene expression assay
POU5F1 (OCT4)	Hs03005111_g1
SOX2	Hs01053049_s1
NANOG	Hs02387400_g1
PROM1 (CD133)	Hs01009261_m1
KLF4	Hs00358836_m1
EPCAM	Hs00158980_m1
MYC	Hs00905030_m1
CCNA2	Hs00996788_m1
CCNE1	Hs01026536_m1
CCND1	Hs99999004_m1
CCNG1	Hs00171112_m1
CDKN1A	Hs00355782_m1
CDKN2A	Hs99999189_m1
CDKN2B	Hs00793225_m1
FOXM1	Hs01073586_m1
FOXO1	Hs01054576_m1
FOXO3A	Hs00921424_m1
AKT1	Hs00178289_m1
AKT2	Hs01086102_m1
AKT3	Hs00987350_m1
TGFBI	Hs00932747_m1
TGFBR3	Hs01114253_m1
huRPLP0	Hs99999902_m1 Endogenous control
Hsa-miR122	TM:002245
RNU6B	TM:001093 Endogenous control

Supplementary Table S1B: List of antibodies and suppliers used

Antibody	Suppliers
Mouse anti-CK8+18	Abcam ab15224
Mouse anti-CK19	Abcam ab7754
Rabbit anti-EpCAM	Abcam ab32392
Mouse anti-CD133	Abcam ab27679
Mouse anti-cMYC	Abcam ab32
Rabbit anti-OCT4	Abcam ab18976
Rabbit anti-SOX2	Abcam ab59776
Rabbit anti-NANOG	Abcam ab21624
Rabbit anti-IGF-1R	Abcam ab131476
Rabbit anti-INSR1/IGF-1R (phospho Y1161)	Abcam ab172965
Mouse anti-Cyclin D1	Abcam ab6152
Mouse anti-AKT1	Cell Signaling #2967
Rabbit anti-AKT (phospho Ser473)	Cell Signaling #9271
Rabbit anti-AKT2	Cell Signaling #2964
Rabbit anti-AKT2 (phospho Ser474)	Cell Signaling #8599
Rabbit anti-AKT3	Abcam ab152157
Rabbit anti FOXO3A	Abcam ab53287
Rabbit anti-FOXO3A (phospho S253)	Abcam ab154786
Rabbit anti-ERK1/2	Cell Signaling #4695
Rabbit anti-ERK1/2 (phospho Thr202/Tyr204)	Cell Signaling #4370
Rabbit anti-p38	Cell Signaling #8690
Rabbit anti-p38 (phospho Thr180/Tyr182)	Cell Signaling #4511
Rabbit anti-βActin (loading control)	Abcam ab8227
Mouse anti-SFPQ (loading control)	Abcam ab11825
